# Identification and development of TRPM4 antagonists to counteract neuronal excitotoxicity

**DOI:** 10.1016/j.isci.2024.111425

**Published:** 2024-11-19

**Authors:** Lars Binkle-Ladisch, Andy Pironet, Andrea Zaliani, Chantal Alcouffe, Daniel Mensching, Undine Haferkamp, Anne Willing, Marcel S. Woo, Alexandre Erdmann, Timm Jessen, Stephen D. Hess, Philip Gribbon, Ole Pless, Rudi Vennekens, Manuel A. Friese

**Affiliations:** 1 Institute of Neuroimmunology and Multiple Sclerosis, University Medical Center Hamburg-Eppendorf, 20251 Hamburg, Germany; 2 Laboratory of Ion Channel Research, Department of Cellular and Molecular Medicine, Katholieke Universiteit Leuven, Campus Gasthuisberg O/N1, Herestraat 49-Bus 802, 3000 Leuven, Belgium; 3 Fraunhofer Institute for Translational Medicine and Pharmacology ITMP, 22525 Hamburg, Germany; 4 Department of Chemistry, Evotec SE, 195 Route D'Espagne, 31036 Toulouse, France; 5 SCIENAMICS GmbH, 24975 Husby, Germany; 6 Evotec Asia Pte Ltd, 79 Science Park Drive, #04-05 Cintech IV, Singapore 118264, Singapore

**Keywords:** Molecular biology, Neuroscience, Molecular neuroscience

## Abstract

Neurodegeneration in central nervous system disorders is linked to dysregulated neuronal calcium. Direct inhibition of glutamate-induced neuronal calcium influx, particularly via N-methyl-D-aspartate receptors (NMDAR), has led to adverse effects and clinical trial failures. A more feasible approach is to modulate NMDAR activity or calcium signaling indirectly. In this respect, the calcium-activated non-selective cation channel transient receptor potential melastatin 4 (TRPM4) has been identified as a promising target. However, high affinity and specific antagonists are lacking. Here, we conducted high-throughput screening of a compound library to identify high affinity TRPM4 antagonists. This yielded five lead compound series with nanomolar half-maximal inhibitory concentration values. Through medicinal chemistry optimization of two series, we established detailed structure-activity relationships and inhibition of excitotoxicity in neurons. Moreover, we identified their potential binding site supported by electrophysiological measurements. These potent TRPM4 antagonists are promising drugs for treating neurodegenerative disorders and TRPM4-related pathologies, potentially overcoming previous therapeutic challenges.

## Highlights

• Identification of 5 TRPM4 lead compounds with nanomolar IC50
• Medicinal chemistry optimized compounds counteract neuronal excitotoxicity
• Insights into TRPM4 antagonist binding by mutational and electrophysiology studies
• Minimal off-target effect and low toxicity suggest promising neuroprotective drugs

## Graphical abstract

**Figure:**
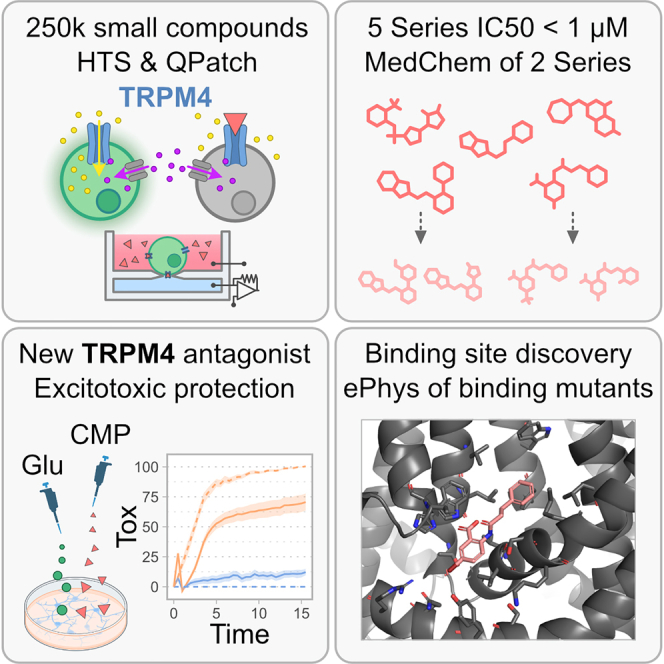

## Introduction

Neurons are characterized by their remarkable electrical excitability and elaborated networks distinguishing them from other cell types. This excitability is facilitated by an electrochemical gradient, primarily established through the activity of the potassium-sodium pump, allowing subsequent ion movement between the cytosol and the extracellular space via ion channels in the plasma membrane.^1^ However, this distinctive feature can become a liability when neuronal excitation surpasses physiological limits in central nervous system (CNS) disorders. Glutamate, the primary excitatory neurotransmitter in the mammalian nervous system, is intricately linked to tightly regulated intracellular calcium levels that are essential for numerous cellular processes.^2^^,^^3^ Specifically, synaptic release of glutamate that activates N-methyl-D-aspartate receptors (NMDAR) is crucial for calcium influx, initiating a cascade of calcium-dependent processes that are, for instance, involved in learning and memory and are required for neuronal survival.^4^^,^^5^ In contrast, pathologically excessive glutamate release can overburden this system, leading to calcium-dependent neuronal damage and cell death known as excitotoxicity.^6^^,^^7^^,^^8^^,^^9^

Several mechanisms contribute to elevated extracellular glutamate levels. Although astrocytes efficiently remove excess glutamate from the extracellular space, they can also release glutamate to modulate neuronal activity.^10^ Excessive neuronal activity may cause glutamate spillover from synaptic sites, activating extra-synaptic NMDARs.^8^ During neuroinflammation, activated microglia^11^^,^^12^^,^^13^ and infiltrating T cells^14^ serve as additional sources of glutamate. Consequently, excitotoxicity is a significant contributor to neuronal cell death in various acute and chronic neurodegenerative disorders such as stroke, Alzheimer’s disease (AD), Parkinson’s disease (PD), amyotrophic lateral sclerosis (ALS), and multiple sclerosis (MS).^15^^,^^16^^,^^17^

Pharmaceutical targeting of NMDARs or glutamate metabolism has yielded unsatisfying outcomes. Interfering with neurotransmitter levels or synaptic transmission poses the risk of severe side effects, complicating therapy development.^18^^,^^19^^,^^20^^,^^21^ An alternative approach involves targeting cytotoxic events downstream of the NMDAR, with the goal of attenuating adverse effects while mitigating the deleterious effects of neuronal excitotoxicity.^22^^,^^23^^,^^24^^,^^25^^,^^26^ In this regard, the transient receptor potential melastatin 4 (TRPM4) channel, which functions as a calcium-activated non-selective cation channel capable of amplifying toxic NMDAR signaling,^24^ has emerged as a promising therapeutic target.

TRPM4 belongs to the evolutionarily conserved family of transient receptor potential (TRP) channels. This family consists of 28 cation permeable channels with six putative transmembrane domains that are grouped into six subfamilies.^27^ The melastatin-related TRP (TRPM) subfamily consists of eight members that can be further divided into four groups.^28^ TRPM4 and the closest homolog TRPM5 distinguish themselves from other members of the TRPM family by the capacity to only conduct monovalent cations. TRPM5 is primarily expressed in pancreatic beta-cells, intestinal and taste cells and is particularly important for taste signaling.^29^ In contrast, TRPM4 is widely expressed throughout the body and is activated by intracellular calcium binding, coordinated by conserved amino acids of the S2 and S3 transmembrane helices.^30^ Channel gating is also sensitive to membrane potential,^31^ phosphoinositide lipids in the plasma membrane^31^^,^^32^ and the local concentration of cytoplasmic ATP.^33^ Sulfonylurea receptor 1 (SUR1) has been proposed as auxiliary subunit of the TRPM4 channel and SUR1-TRPM4 was found to form a complex with aquaporin-4 (AQP4), regulating water influx at the astrocytic endfeet.^34^^,^^35^ Glibenclamide, a US Food and Drug Administration (FDA)-approved oral antidiabetic drug (also known as glyburide), has been shown to block SUR1-associated channels including TRPM4.^36^^,^^37^^,^^38^

TRPM4 also binds to the NMDAR and regulates its subcellular localization. Pharmacological dissociation of the neuronal TRPM4-NMDAR complex or glibenclamide treatment improve the outcome of ischemic stroke-induced brain damage in mice.^24^^,^^38^ Moreover, glibenclamide treatment protects from neurodegeneration in a mouse model of neuroinflammation.^39^ Genetic ablation or antibody blocking of TRPM4 was shown to be beneficial in mouse models of MS and ischemic stroke without detectable impairments due to loss of TRPM4 function.^35^^,^^39^^,^^40^^,^^41^ Beyond neurological disorders,^39^^,^^42^^,^^43^ TRPM4 has been associated with a variety of conditions, including genodermatosis,^44^ cardiac,^45^^,^^46^^,^^47^^,^^48^^,^^49^^,^^50^ and immune-mediated^51^^,^^52^^,^^53^^,^^54^ diseases. In all of these cases, the primary underlying cause is an increase in TRPM4 function by gain-of-function mutations, *de novo* expression or up-regulation of TRPM4 expression. Consequently, antagonizing TRPM4 function is a promising strategy for the treatment of various diseases, in particular neurodegeneration.

Until today there are no TRPM4 drugs approved for human use except glibenclamide and meclofenamate, which suffer from low affinity and lack of specificity.^45^^,^^55^^,^^56^ Only recently, advancements have led to the development of anthranilic acid derivatives, including 4-chloro-2-[[2-(2-chlorophenoxy)acetyl]amino]benzoic acid (CBA), with better antagonistic affinity.^57^ However, none of these anthranilic acid derivatives have been successfully applied in preclinical models.

Here, we used high-throughput screening to identify TRPM4 antagonists, unveiling five lead series with nanomolar IC50 values. Subsequent derivatization and testing led to the development of two series of compounds that reduce neuronal excitotoxicity, underscoring TRPM4 as therapeutic target. By an analysis of related channel structures, we deduced a specific binding site for these compounds on TRPM4, substantiated by mutational analysis and electrophysiological studies. Our findings provide a robust foundation for further optimization of these potent and highly specific antagonists toward clinical use.

## Results

### Identification of TRPM4 antagonists through high-throughput screening

**Figure 1:**
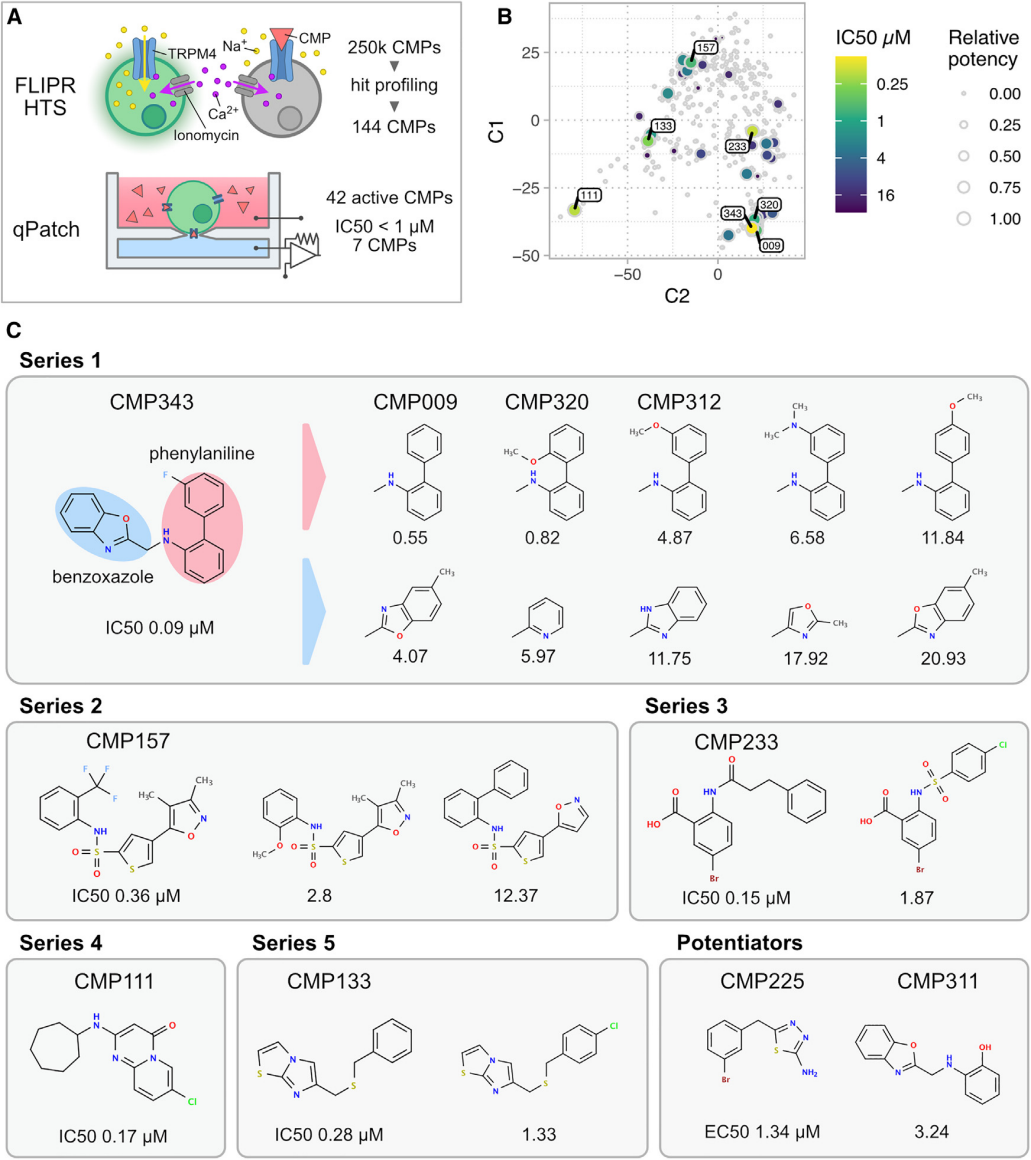
Identification of five lead TRPM4 antagonist series by high-throughput screening (A) Schematic illustration of the high-throughput screening (HTS) workflow for the identification of TRPM4 antagonists. The HTS involved screening a library comprising 256,286 small molecules. Active compounds were identified using a kinetic FLIPR assay based on membrane-potential sensitive dyes followed by subsequent validation of their activity and determination of IC50 values with an automated patch clamp system (QPatch). (B) Multidimensional scaling (MDS) analysis presenting the distribution of 357 compounds identified during the high-throughput screening. The respective IC50 values determined by QPatch measurements are represented, with compounds exhibiting IC50 values below ≤1 μM highlighted. (C) Assignment of lead compounds and active orthologs to five distinct compound series summarizing chemical features of the identified TRPM4 antagonists. Also see Figures S1–S3.

To screen for TRPM4 antagonists, we used HEK293 cells that express human (hs) TRPM4. We measured hsTRPM4-mediated ion flux and assessed its inhibition by small molecules, using a membrane potential-sensitive blue dye. Activating of hsTRPM4 was achieved by an ionomycin-mediated increase of intracellular calcium levels (Figure 1A). This assay was customized to a high-throughput screening (HTS) compatible 384-well microplate format and was read out using a fluorometric imaging plate reader (FLIPR) to acquire kinetic data.

In our primary screen, we evaluated a library of lead-like compounds, totaling 256,286 substances, at a concentration of 10 μM. The hit criterion was set as a minimum of 34% reduction in TRPM4 activity for compounds exhibiting an acceptable quantitative estimate of drug-likeness (QED). Compounds known as frequent hitters were excluded from further analysis, resulting in the nomination of 7,633 TRPM4 antagonists, constituting a hit rate of 3%. Subsequent automated cluster-based selection narrowed down the pool to 5,040 compounds, which underwent counter screening.

The counter screening phase involved the inclusion of hsTRPM5-expressing HEK293 cells, as closest homologue, and the parental HEK293 cell line to evaluate selectivity. This screening confirmed the activity of 4,420 hsTRPM4 antagonists (69% of the hit population of the primary screen), among which 529 compounds (12%) showed non-desired activity on hsTRPM5 or the parental cell line. Based on categorization of biological activity, mass spectrometry quality control, and aspects of medicinal chemistry, we prioritized 144 selective hsTRPM4 compounds. These compounds were then subjected to automated patch clamp electrophysiology measurements (QPatch^58^) to evaluate their activity profiles and IC50 values (Figures 1A, S1, and S2).

Among these, we confirmed the on-target efficacy of 42 compounds, seven of which exhibited sub-micromolar IC50 values. Utilizing multidimensional scaling (MDS) visualization, we analyzed compound similarities and revealed that these seven compounds could be assigned to five distinct groups, each comprising one to ten closely related active members. From these groups, five compounds with the lowest IC50 values were selected as lead compounds, forming the basis of five potential compound series (Figure 1B).

Further exploration of lead compound 1 (CMP343, IC50 = 0.09 μM) derivatives identified ten related active compounds with modifications in the phenylalanine or benzoxazole residues. We also identified two active derivatives of lead compound 2 (CMP157, IC50 = 0.36 μM), and one each for lead compound 3 (CMP233, IC50 = 0.15 μM) and lead compound 5 (CMP133, IC50 = 0.28 μM). Lead compound 4 (CMP111, IC50 = 0.11 μM) did not yield any potent derivatives. Two active compounds (CMP225, EC50 = 1.34 μM; CMP311, EC50 = 3.24 μM) unexpectedly showed potentiator activity, with CMP311 being associated with Series 1 (Figures 1C and S3). Series 3 compounds were recognized as anthranilic acid orthologs, thus belonging to the group of known TRPM4 antagonists such as flufenamic acid (FFA)^59^ and CBA.^57^ Compounds of the remaining series represent previously undescribed classes of TRPM4 antagonists.

### Development and assessment of compound Series 1 and 3

**Figure 2:**
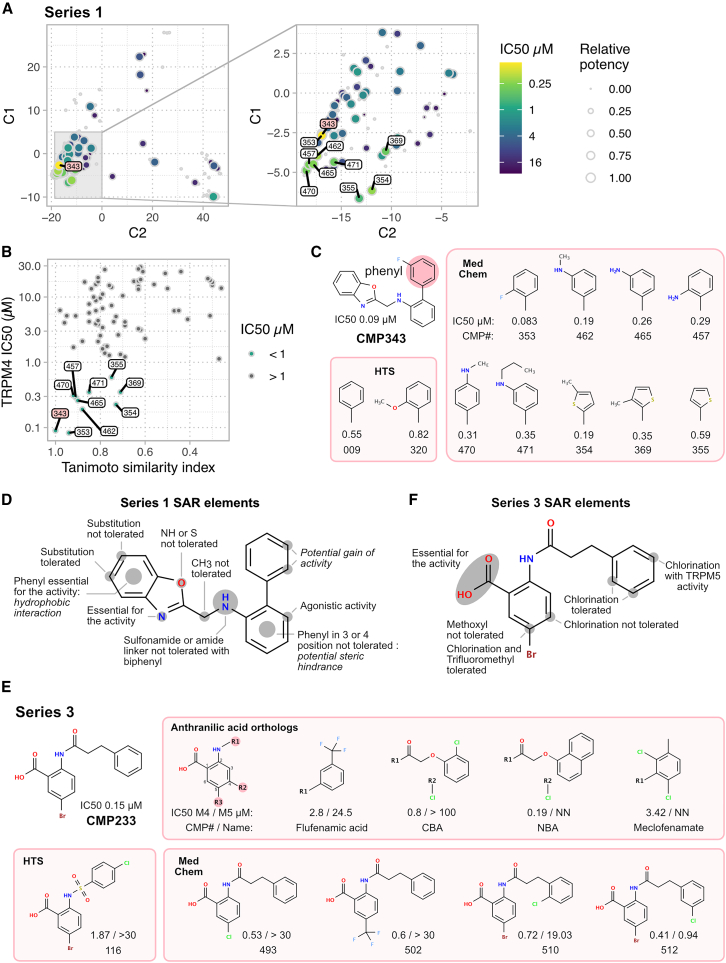
Medicinal chemistry of lead compounds and structure-activity relationship (A) Multidimensional scaling (MDS) analysis of 152 derivatives from Series 1 with IC50 values color coded for 78 active compounds. (B) Illustration of the correlation between lead similarity and IC50. (C) Series 1 compounds identified through high-throughput screening (HTS) and medicinal chemistry (MedChem), with IC50 values below 1 μM. The focus lies on modifications of the phenyl residue that maintain IC50s in the nanomolar range. (D) Exploration of structure-activity relationship (SAR) elements, determined through MedChem optimization in Series 1. (E) Comparison of Series 3 compounds with known TRPM4 antagonists of the anthranilic acid ortholog family. (F) Exploration of SAR elements determined through MedChem optimization in Series 3. Also see Figures S2–S5.

Based on our initial discoveries of several active derivatives in Series 1 and the similarities of Series 3 to known TRPM4 anthranilic acid orthologue inhibitors, we selected these two series for further optimization by medicinal chemistry. In Series 1, we synthesized 152 new compounds and assessed their IC50 values using the QPatch assay. About half of the derivatives, 78 compounds, showed inhibitory activity. MDS analysis indicated that these compounds predominantly occupy a narrow chemical space (Figure 2A). From this analysis, we identified nine new compounds in Series 1 with sub-micromolar IC50 values, demonstrating close chemical relationships (Figures 2B and S2). Together with three compounds of the initial HTS, Series 1 comprises a total of 12 antagonists with sub-micromolar IC50 value. These derivatives either contain a modified phenyl residue or a substitution of the benzene by thiophene (Figure 2C). The medicinal chemistry optimization efforts allowed us to derive the structure-activity relationship (SAR) of Series 1. Our findings, consistent with the MDS analysis, delineate a constrained but clearly defined SAR, indicating a limited spatial availability within the TRPM4-binding pocket. Notably, certain compounds within Series 1 functioned as potentiators, despite minimal alterations to their backbone structure in some instances (Figures 1C, S3, and S5). This intriguing observation suggests the potential binding of Series 1 compounds to an allosteric site. Additionally, two compounds from this series exhibited activity on both TRPM4 and TRPM5, with IC50 values for CMP381 of 1.13 μM and 3.87 μM, and for CMP384 of 4.56 μM and 18.34 μM, respectively. This observation suggests that Series 1 compounds may share a common binding site on both channels (Figures 4 and S5).

For Series 3, we performed a targeted medicinal chemistry optimization and evaluated the active compounds against established anthranilic acid orthologs. Notably, Series 3 compounds showed a distinct profile from known antagonists; they did not tolerate chlorination at position 4 but did at position 5, which also accepted bromination and trifluoromethylation. This indicates greater steric flexibility of Series 3 compounds. Furthermore, we expanded the scope of acceptable modifications for anthranilic acid TRPM4 antagonists to encompass benzene sulfonamide and phenylpropane derivatives. Notably, phenylpropane demonstrated sub-micromolar IC50 values showing promising potential for further optimization (Figures 2E and 2F). CMP512 displayed activity on both TRPM4 and TRPM5, with IC50 values of 0.41 μM and 0.94 μM, respectively. This observation suggests a potential shared binding site for Series 3 compounds on both channels. Similar to Series 1, we also identified compounds within Series 3 functioning as potentiators, indicating the possibility of Series 3 compounds binding to an allosteric site (Figures S3–S5).

We next used *in vitro* assays to test absorption, distribution, metabolism, excretion and toxicity (ADMET) parameters for selected compounds. We assessed compound absorption using parallel artificial membrane permeability (PAMPA) and Caco-2 cell permeability assays (cut-off criterion >2 × 10^−6^ cm s^−1^). Compound toxicity was evaluated by measuring inhibition of cytochrome P450 enzymes (CYP), the potassium channel human Ether-a-go-go Related Gene (hERG), and TRPM5, with all parameters meeting acceptable criteria. By contrast, high rate of hepatocyte and microsome clearance suggested metabolic instability (Table 1). To assess potential off-target activity of our compounds that could be relevant for safety in humans, we conducted comprehensive *in vitro* pharmacology using the commercially available and widely used SafetyScreen44 interaction panel^60^ covering eight protein families. Of note, only compounds from Series 1 showed significant off-target effects, with the most pronounced being a 78% inhibition of the norepinephrine transporter (Tables 2 and 3).

**Table 1:** *In vitro* ADMET profile of Series 1 compounds

CID	TRPM4 IC50 μM	Inhib. at highest conc. %	TRPM5 IC50 μM	hERG IC50 μM	Hep. clearance L/h/kg	Microsomes (m) clearance L/h/kg	Microsomes (h) clearance L/h/kg	Cyp IC50 μM	Caco-2 perm. 10^−6^ cm/s	PAMPA perm. 10^−6^ cm/s
CMP343	0.09	48.76	>10	>30	19.3	>54	34.2	2C9 = 32.2, 2D6>50, 3A4>50, 1A2 = 3.2, 2C19 = 3.5, TDI 3A4>50	–	–
CMP353	0.08	12.46	>10	>10	33.7	>54	36.3	2C9 = 25.9, 2D6>50, 3A4>29.6, 1A2 = 1, 2C19 = 3.3	–	low sensitivity
CMP354	0.19	12.2	>10	>10	17.6	>54	27.6	2C9 = 25, 2D6>50, 3A4>50, 1A2 = 2.4, 2C19 = 3.7, TDI 3A4>50	–	–
CMP355	0.59	22.74	>10	>10	17.6	>54	33.5	2C9 = 14.9, 2D6>50, 3A4>50, 1A2 = 1.3, 2C19 = 6.2, TDI 3A4>50	–	–
CMP369	0.35	24.24	>10	>10	20.8	>54	36	2C9 = 25,4 2D6>50, 3A4>50, 1A2 = 27,2 2C19 = 11,4	52	–
CMP457	0.29	24.69	>30	24.91	–	54	14.6	2C9 = 21.3 2D6>50, 3A4 = 10.6, 1A2 = 37.8 2C19 = 6.8	210	37.4
CMP462	0.19	39.14	>30	8.27	–	54	18.12	2C9 = 18 2D6>50, 3A4 = 19.2, 1A2 = 3.3 2C19 = 4	233	25
CMP465	0.26	23.36	>30	26.57	–	23.6	12.96	2C9 = 17.6 2D6>50, 3A4 = 4.5, 1A2 = 2.3 2C19 = 3.2	189	37.9
CMP470	0.31	79.9	>30	10.6	–	54	13.5	2C9 = 9.8 2D6>50, 3A4 = 2.9, 1A2 = 1.3 2C19 = 1.2	–	22
CMP471	0.35	89.7	>30	2.55	–	–	–	2C9 = 17.2 2D6>50, 3A4>50, 1A2 = 16.7 2C19 = 14.7	–	no detection

CID = Compound ID; PAMPA = Parallel Artificial Membrane Permeability Assay.

**Table 2:** *In vitro* pharmacology profile of CMP 157, 233, 343 and 354 Only inhibitions above 50% relative to the reference compound are shown and considered active

CID	Assay	Conc. (μM)	Inhibition (%)	Ref. CMP
343	kappa (h) (KOP) (agonist radioligand)	5	63	U50488
343	5-HT2A (h) (agonist radioligand)	5	51	(±)DOI
343	5-HT2B (h) (agonist radioligand)	5	70	(±)DOI
343	norepinephrine transporter (h) (antagonist radioligand)	5	78	protriptyline
343	dopamine transporter (h) (antagonist radioligand)	5	70	BTCP
343	COX1(h)	5	56	Diclofenac
354	5-HT2A (h) (agonist radioligand)	5	73	(±)DOI
354	5-HT2B (h) (agonist radioligand)	5	65	(±)DOI
354	norepinephrine transporter (h) (antagonist radioligand)	5	74	protriptyline
354	dopamine transporter (h) (antagonist radioligand)	5	64	BTCP

KOP = Kappa-type opioid receptor; 5-HT2A/B = 5-hydroxytryptamine receptor 2A/B; COX1 = Cytochrome *c* oxidase subunit 1; CID = Compound ID; Ref. CMP = Reference compound; (±)DOI = 2,5-Dimethoxy-4-iodoamphetamine racemate; BTCP = 1-[1-(1-benzothiophen-2-yl)cyclohexyl]piperidine.

**Table 3:** Protein families tested for *in vitro* pharmacology profile

Family	*n*
GPCR	24
Ion Channel	8
Kinase	1
Lipid Metabolism	2
NHR	2
Neurotransmitter Metabolism	2
PDE	2
Transporter	3

GPCR = G protein-coupled receptors; NHR = Nuclear hormone receptors; PDE = Phosphodiesterases.

These results show the need for improvements in metabolic stability while indicating favorable membrane permeability, minimal toxicity and off-target liabilities, suggesting a promising safety profile for future applications in humans.

### Evaluation of neuroprotective properties and *in vivo* pharmacokinetics

**Figure 3:**
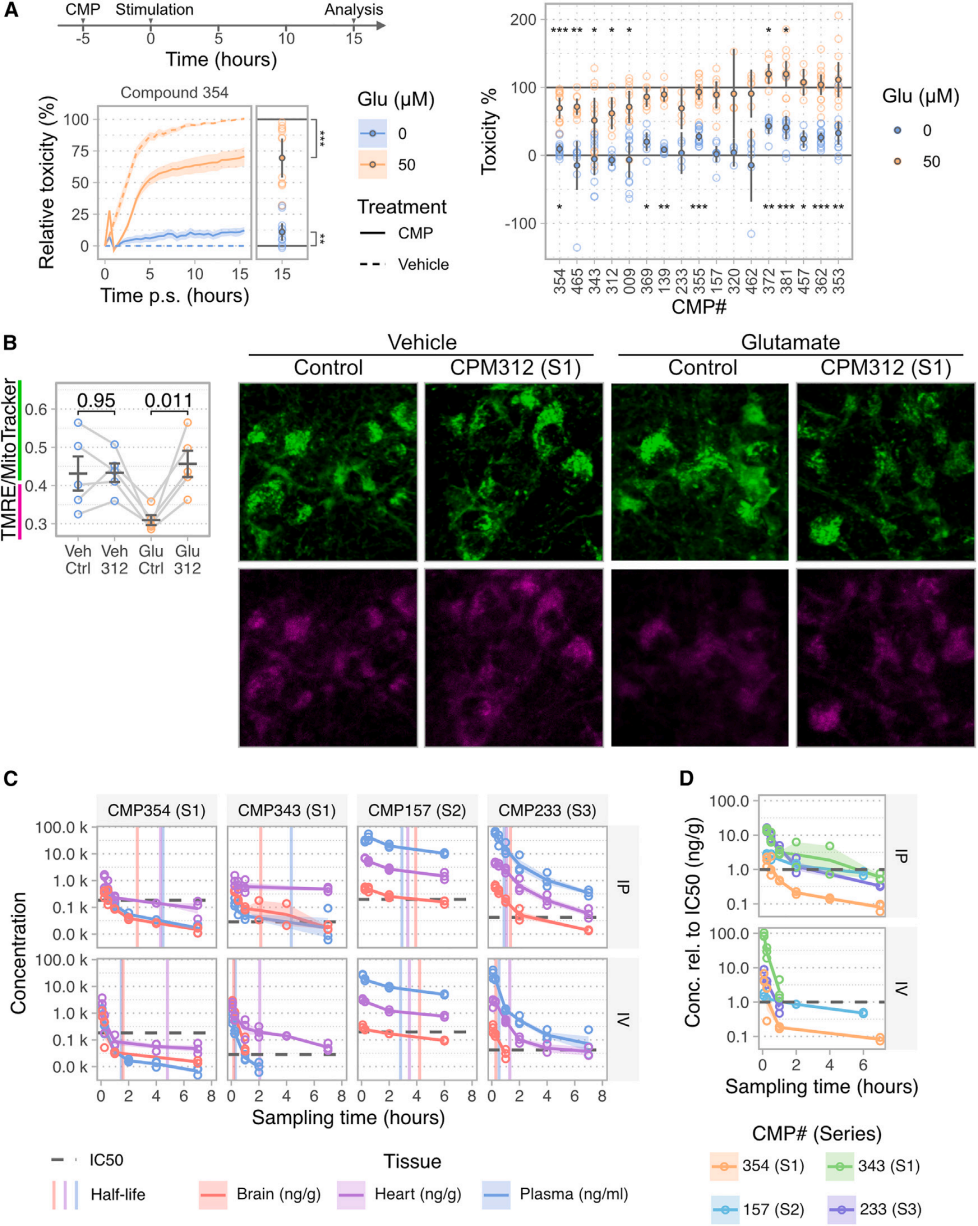
Neuroprotective *in vitro* evaluation and *in vivo* pharmacokinetics (A) *In vitro* evaluation of compound activity on glutamate-induced excitotoxicity in mature primary neuronal cultures. Neuronal cultures were treated with 5 μM of the specified compound or vehicle control 5 h prior to glutamate stimulation, assessing compound rescue activity and toxicity relative to minimal (Vehicle +0 μM Glu) and maximal (Vehicle +50 μM Glu) excitotoxicity. Time course of a representative compound with virtual endpoint at 15 h post stimulation and assay window definition (left). Toxicity of compound treated cultures with and without glutamate stimulation (right). Data are represented as mean and 95% confidence interval; *n* ≥ 4; ∗*p* < 0.05, ∗∗*p* < 0.01, ∗∗∗*p* < 0.001; One-sample Wilcoxon signed-rank test. (B) Assessment of CMP312 on mitochondrial integrity after glutamate-induced excitotoxicity. Mitochondrial membrane potential was measured by the mean fluorescence intensity of TMRE normalized to MitoTracker. Data are represented as mean ± SEM; *n* = 5; paired *t*-test. (C) *In vivo* pharmacokinetics of four selected compounds. Time course of compound concentration in the brain, heart, and plasma after a single dose via intraperitoneal (i.p.) or intravenous (i.v.) application. Data are represented as mean ± SEM. Half-life estimates by non-compartmental analysis are shown as vertical line and IC50 value as dashed horizontal line. (D) Compound concentration in the brain relative to respective IC50 concentrations after a single dose via intra-peritoneal or intra-venous routes. Also see Figure S6.

To evaluate the *in vitro* efficacy of our developed compounds, we employed primary mouse neuronal cultures (PNCs) and conducted glutamate-induced excitotoxicity assays. Cultures were pre-treated with respective Series 1 and Series 3 compounds before glutamate exposure, and neuronal viability was assessed 15 h post-stimulation with glutamate. Notably, several compounds from Series 1 and the lead compound from Series 3 significantly reduced glutamate excitotoxicity ranging from 5% to 50%, confirming their biological activity. The activity of additional compounds (CMP139, 353, 355, 362, 369, 372, 381 and 457) might be masked by their observed toxicity at the tested concentrations of 5 μM (Figures 3A and S6).

As an example, we further validated the efficacy of Series 1 by utilizing CMP312, which showed both non-toxicity and potent protective effects in the excitotoxicity assay (Figures 1C and 3A). We assessed its ability to inhibit the deterioration of mitochondrial membrane potential (ΔΨm) in response to glutamate-induced excitotoxicity. The ratio between the ΔΨm-dependent dye tetramethylrhodamine ethyl ester (TMRE) and the mitochondrial dye MitoTracker was quantified for this purpose. As expected, treatment with CMP312 mitigated the glutamate-induced decrease in ΔΨm, thus affirming its protective properties (Figure 3B).

Next, to assess *in vivo* availability of the Series 1–3 compounds, we evaluated drug metabolism and pharmacokinetics (DMPK). We measured compound concentrations in plasma, heart, and brain at several time points after intraperitoneal (i.p.) or intravenous (i.v.) single-dose injections. Especially, compounds from Series 2 and 3 showed high levels in plasma and heart, indicating good bioavailability. Conversely, in accordance with our ADMET studies, Series 1 compounds exhibited a rapid decrease in concentrations in all organs analyzed, with an estimated 50% bioavailability (Figure 3C; Table 4). Focusing on compound concentrations in the brain as the target organ of neurodegeneration, we found that the concentrations of all compounds remained below their respective IC50 values 4 h after injection (Figure 3D).

**Table 4:** Summary of *in vivo* pharmacokinetic properties

	Route	CMP233	CMP157	CMP354	CMP343
IC50	–	0.15 μM	0.63 μM	0.19 μM	0.09 μM
Bioavailability	–	95%	104%	55%	57%
Plasma half-life	IP	0.9 h	2.9 h	4.5 h	4.3 h
Heart half-life	IP	1.1 h	3.3 h	4.3 h	15.3 h
Brain half-life	IP	1.4 h	3.9 h	2.6 h	2.1 h
Dose	IP	10 mg kg^−1^	5 mg kg^−1^	10 mg kg^−1^	10 mg kg^−1^
Plasma half-life	IV	0.5 h	2.8 h	1.5 h	0.3 h
Heart half-life	IV	1.3 h	3.5 h	4.8 h	2.1 h
Brain half-life	IV	0.3 h	4.2 h	1.6 h	0.2 h
Dose	IV	3 mg kg^−1^	2.5 mg kg^−1^	3 mg kg^−1^	3 mg kg^−1^

Taken together, these data demonstrate the high potential of our compounds to alleviate excitotoxicity in neurons. However, they also indicate the necessity for improvements in stability for *in vivo* application.

### Discovery of the compound binding site

**Figure 4:**
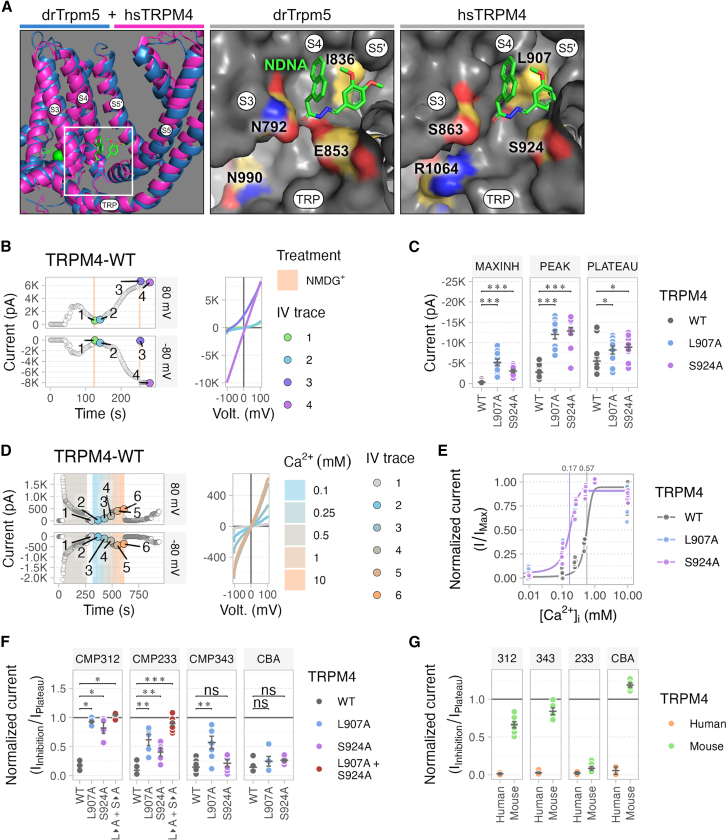
Electrophysiological analysis of proposed compound binding site (A) Proposed compound binding site. (left) Aligned superimposition of drTRPM5 structure (blue) in complex with its antagonist NDNA (green) and hsTRPM4 (magenta). Transmembrane helices (S) and the TRP domain are labeled. drTrpm5 binding pocket of NDNA (middle) and the corresponding pocket in hsTRPM4 (right). Divergent amino acids are labeled. (B and C) Whole cell voltage-clamp analysis of basic electrophysiological properties of HEK293T cells expressing wild-type (WT) or mutant hsTRPM4. (B) Representative time course of current levels at −80 and +80 mV recorded in hsTRPM4-expressing HEK293T cells. TRPM4 currents were elicited upon patch rupture by 100 μM CaCl_2_, loaded in the pipette solution. Extracellular Na^+^ was replaced by equimolar N-methyl-D-glucamine (NMDG^+^) to identify TRPM4 currents. Individual current-to-voltage relationship measured in the same hsTRPM4-expressing HEK293T cells are shown next to the representative time course. IV trace numbers indicate the respective time points of the measurement to calculate peak current, maximal inhibition and plateau current. (C) Quantification of the peak current, maximal inactivated current and the steady-state plateau current between WT and hsTRPM4-L907A and hsTRPM4-S924A mutants. Data are represented as mean ± SEM. (D and E) Assessment of Ca^2+^ sensitivity of WT and mutant hsTRPM4. (D) Representative time course of current levels at −80 mV and +80 mV recorded in hsTRPM4-expressing HEK293T cells using the inside-out patch-clamp technique. TRPM4 currents were activated by 500 μM Ca^2+^ and deactivated by 10 mM EGTA. Individual current-to-voltage relationship measured in the same hsTRPM4-expressing HEK293T cell are shown next to the representative time course. (E) The Ca^2+^ concentration-to-current relationship of WT and hsTRPM4-L907A and hsTRPM4-S924A mutants. Likelihood maximization was used to estimate the parameters for the sigmoidal fitting curves with y-maximum, slope and midpoint of 0.94, 8.05 and 0.55 (WT); 0.90, 18.00 and 0.16 (L907A); 0.91 and 17.57 (S924A). (F) Quantification of relative inhibition in whole cell voltage-clamp configuration of WT and mutant hsTRPM4 by Series 1 compounds (CMP312, CMP343), Series 3 compound (CMP233) and CBA. Data are represented as mean ± SEM. (G) Comparison of relative inhibition in whole cell voltage-clamp configuration between mouse and human TRPM4. C: *n* ≥ 10; F: *n* ≥ 5; ∗*p* < 0.05, ∗∗*p* < 0.01, ∗∗∗*p* < 0.001; Wilcoxon rank-sum test. Also see Figures S7–S10.

Having detected potent *in vitro* efficacy of our compounds to inhibit glutamate-induced excitotoxicity, we aimed to identify their TRPM4 binding site, which is crucial for further compound optimization. To this end, we analyzed the known binding sites of small molecule activators and inhibitors within the TRP channel family. *In silico* comparison revealed that binding of small molecules typically occurs in cavities above the Ca^2+^-binding site, as seen in collared flycatcher (cf.) TRPM8,^61^ or involving the transmembrane helices S3–S4 and the pore forming domain, with helix 3 being involved in Ca^2+^-binding, as observed in human TRPV1,^62^^,^^63^^,^^64^ rabbit TRPV5,^65^ human TRPA1^66^ and zebrafish (dr) TRPM5.^67^ Notably, the interaction of N′-(3,4-dimethoxybenzylidene)-2-(naphthalen-1-yl)acetohydrazide (NDNA) with drTRPM5, due to its structural similarity to TRPM4 and comparable size to our compounds, offered valuable insights. A structural comparison, aligning NDNA-bound drTRPM5 and hsTRPM4, suggested the conservation of a hydrophobic binding cavity in hsTRPM4. The cavity appeared more spacious toward the cytosolic interface, attributed to the substitution of E835 and N792 in drTRPM5 by S863 and S924 in hsTRPM4 respectively. Furthermore, the cytosolic interface of the cavity is lined by the TRP domain, crucial for channel gating, where N990 in drTRPM5 is substituted by R1064 in human TRPM4 and S1060 in mouse TRPM4 (Figures 4A and S7).

We employed the structure-based method DoGSite to predict binding pockets and used the support vector machine-based DoGSiteScorer to assess the druggability of these predicted sites. In the Ca^2+^-bound hsTRPM4,^30^ a binding pocket with a druggability score of 0.73, hydrophobicity of 0.51 and a volume of 329 Å^3^ was predicted, adequately sized to accommodate our compounds. Similarly, in the ligand-free (apo) drTRPM5, a binding pocket with a druggability score of 0.73, hydrophobicity of 0.49 and a volume of 350 Å^3^ was predicted (Figures 4A and S7). In drTRPM5, Ca^2+^ binding triggers the relative movement between the S4-S5 linker and the TRP domain, facilitating the repositioning of the pore forming S5-S6 helices and channel opening. Consequently, the binding site of NDNA is well-positioned to interfere with this movement, and NDNA-binding stabilizes Ca^2+^-bound drTRPM5 in an apo-like closed state.^67^ In TRPM4, Ca^2+^ binding is thought to precede voltage-dependent opening^31^ with gating mechanisms believed to operate similarly.^68^ *In silico* docking of our compounds to TRPM4 shows a binding configuration (Figure S7) that we propose is comparable to the binding of NDNA to TRPM5.

To validate our hypothesis, we genetically engineered hsTRPM4 mutants with alanine substitutions (L907A and S924A) aimed at disrupting this proposed compound binding site. We expected L907A, corresponding to I836 in drTRPM5, to reduce the hydrophobic interactions within the cavity. Alongside, we anticipated that S924A, corresponding to E853 in drTRPM5, would affect compound binding in a manner similar to NDNA in drTRPM5 (Figure 4A). Initially, we assessed the basic properties of the hsTRPM4 mutants using whole-cell patch clamp electrophysiology. Both mutants, L907A and S924A, exhibited the typical time course of activation for WT TRPM4, as was described before.^69^ Notably, both mutants appeared as gain-of-function variants, exhibiting an increase in current density at the initial peak and at the plateau during steady state (Figures 4B and S8A). These findings correlated with a 3.5-fold increase in Ca^2+^ sensitivity of the mutants (Figures 4C and S8B).

Subsequently, we compared the inhibition of hsTRPM4 wildtype and L907A and S924A mutants by selected compounds (CMP312, CMP343, CMP233) alongside the known TRPM4 antagonist CBA.^57^ All tested compounds demonstrated significantly reduced current inhibition in at least one hsTRPM4 mutant variant, whereas inhibition by CBA remained unaffected. Moreover, in the L907A/S924A double mutant the affinity for CMP312 and 233 is completely abolished (Figures 4D and S9).

Since it has been reported that CBA inhibits human TRPM4 efficiently but not mouse TRPM4,^70^ we also evaluated the species specificity of our compounds. We assessed the activity of our compounds and CBA on both human and mouse TRPM4 using the inside-out patch clamp configuration. Series 1 compounds (CMP343 and CMP312) showed reduced activity on mouse TRPM4 compared to human TRPM4, with currents of human TRPM4 almost completely blocked. In contrast, the Series 3 compound (CMP233) was highly effective in both species, unlike the anthranilic acid ortholog antagonist CBA, which exhibited no activity on mouse TRPM4 (Figures 4E and S10). Together, these results strongly support the interaction of our compounds with the proposed binding site located in a small pocket within the transmembrane region between Ca^2+^-binding and pore-forming domain and offer insights into species-specific activities of these compounds.

## Discussion

Halting neurodegeneration in CNS disorders is an unmet medical need, particularly in aging societies^15^^,^^71^^,^^72^^,^^73^^,^^74^. It is now increasingly recognized that many neurodegenerative disorders share common molecular pathways leading to neuronal cell death. One predominant pathway is the excessive accumulation of calcium in neurons, which can be triggered by excitotoxicity.^75^^,^^76^ TRPM4 emerges as a promising target to counteract neuronal demise due to its interaction with NMDAR and downstream signaling pathways. To discover human TRPM4 antagonists, we conducted a high-throughput screening of a comprehensive small molecule library, yielding a hit rate of 3%, well within the anticipated range.^77^ The active compounds were subjected to validation through QPatch electrophysiology, leading to the identification of five distinct lead series, each exhibiting sub-micromolar IC50 values.

For further medical chemistry optimization, we focused on Series 1 and noted that most modifications led to activity loss, with only 9 out of 152 compounds exhibiting sub-micromolar IC50 values. Our comprehensive SAR analysis indicated that modifications were primarily tolerated within the phenyl residue, suggesting a restricted binding pocket with limited space for alterations. However, acceptable modifications of the phenyl residue present opportunities for further optimization for improved DMPK properties.

Series 3 belongs to the well-established group of anthranilic acid ortholog TRPM4 inhibitors, alongside FFA,^59^ CBA,^57^ and meclofenamate.^45^ We introduced the phenylpropane group as an accepted moiety for the amine group of the anthranilic acid, in conjunction with bromination of the anthranilic acid at position 5. Limited medicinal chemistry optimization also supported chlorination and trifluoromethylation but unlike CBA, Series 3 does not accommodate chlorination at position 4, suggestion a distinct binding conformation. Modifications at position 5 of the anthranilic acid may offer more flexibility for further enhancements, while the carboxyl group remains essential for the activity.^57^ Remarkably, compound 512 demonstrates activity with nanomolar IC50 values not only on TRPM4 but TRPM5, indicating a common binding site, with channel specificity modulated by the phenylpropane group.

Biological activity assays conducted on Series 1 and 3 compounds revealed up to a 50% reduction in excitotoxicity, though not all compounds were active, and some even demonstrated toxic effects on neurons. However, we used comparably high concentrations of 5 μM to accommodate for potential compound activity loss. Importantly, TRPM4 deletion does not result in detrimental effects *in vivo*^39^^,^^41^^,^^51^^,^^52^^,^^54^^,^^78^ or in neuronal cultures,^39^ suggesting that TRPM4 blockade itself is unlikely to cause cytotoxicity. Therefore, the observed neurotoxic effects are most likely due to metabolites of specific derivatives, as these effects were not seen with all compounds. Uncoupling NMDARs from TRPM4 or deleting TRPM4 has been shown to exert neuroprotective effects in both *in vitro* models and stroke mouse models,^24^ likely due to alterations in NMDAR signaling. Mechanistically, it remains unclear whether these changes are driven by the subcellular localization of NMDARs at TRPM4-rich sites or by TRPM4 activity in close proximity to NMDARs. However, our observed neuroprotective effects against excitotoxicity suggest that TRPM4 activity near NMDARs may indeed enhance NMDAR-mediated cytotoxic signaling.

ADMET profiles of our compounds indicated potential for rapid metabolization, possibly affecting activity and generating toxic byproducts. The lack of off-target activities on hERG channels and *in vitro* pharmacology profiles are promising and consistent with absence of toxicity during *in vivo* pharmacokinetic assessments. However, the compounds exhibited low half-lives regardless of the route of administration. This was especially notable in the brain, indicating the necessity for further metabolic stability improvements before conducting *in vivo* studies of neurodegenerative disorders. *In vivo* exploration in mouse models will additionally be hampered by low potency of some compounds on mouse TRPM4. Nevertheless, the exposure levels reached by compounds in the heart are adequate to warrant testing in models of heart disease at adequate dosing, such as those resulting from TRPM4 gain-of-function mutations.

Upon investigating potential binding pockets of our TRPM4 antagonists through comparative analyses within the TRP channel family, we identified two primary binding sites: one located directly above the Ca^2+^-binding site enclosed by the transmembrane helices S1–4,^30^^,^^79^ and another opposite of the S3-4 domain,^67^ situated near the TRP and pore-forming domains. The latter site is also utilized by NDNA for antagonizing the closely related TRP channel TRPM5, making it a promising choice for further investigation. Given the narrow SAR of Series 1 and its compliance with the space limitation of this site, we explored alanine substitutions of hsTRPM4 L907 and S924, corresponding to drTRPM5 I836A and E853A, respectively.^67^ In drTRPM5, these modifications preserved channel functionality but reduced sensitivity to NDNA. Notably, both TRPM4 mutants exhibited an increase in current density, coupled with a 3-fold rise in Ca^2+^ sensitivity. This underscores the close proximity of the compound binding site to TRPM4’s Ca^2+^-binding site and potential interaction between these sites. Crucially, the affinity of these variants for compounds from Series 1 and 3, but not for CBA, was significantly reduced, indicating that L907 and S924 are involved in the binding compounds from both series.

In drTRPM5, substitution of L833A and W869A, corresponding to V904 and W940 in hsTRPM4, led to agonistic activity of NDNA.^67^ Similarly, in TRPV1, the vanilloid agonists resiniferatoxin,^80^ and in TRPA1, the agonist GNE551 ^66^, bind to a pocket homologous to the NDNA-binding site. These findings suggest that this common binding site indeed possesses allosteric properties, wherein minor changes can lead to a switch from antagonistic to agonistic activity and vice versa. Consistently with binding to the proposed site, our medicinal chemistry efforts led to the discovery of TRPM4 potentiators in both Series 1 and 3 (Figure 1C and S5).

When comparing compound activities between mouse and human TRPM4, we observed that Series 1 (CMP312 and 343) is significantly less effective on mouse TRPM4, whereas Series 3 (CMP233) is active on both species, and CBA only affects human TRPM4 as reported previously.^70^ The proposed binding site exhibits only three different amino acids between human and mouse TRPM4. While V904, corresponding to L900 in mouse TRPM4, leads to minor changes at the top of the hydrophobic pocket, its influence may not be significant. However, S868 and R1064, corresponding to T859 and S1060 in mouse TRPM4, respectively, are positioned at the cytosolic interface of the proposed binding pocket (Figure S7). We hypothesize their involvement in binding or modulating accessibility to compounds from Series 1. Additionally, they may impede the binding of CBA, particularly with chlorine at position 4 of the anthranilic acid.

CMP233 from Series 3 exhibits one of the highest affinities among anthranilic acid ortholog inhibitors, coupled with increased flexibility for subsequent structural modifications to improve its pharmacokinetic properties. *In vitro* pharmacological assessments indicate no off-target binding, and the bioavailability in the heart appears sufficient for treatment studies in mouse models of TRPM4-related cardiac diseases.^45^^,^^48^ To our knowledge, Series 1 contains the most potent human TRPM4 antagonist to date with an IC50 of 83 nM and we have delineated a well-defined SAR that will guide further development necessary to improve metabolic stability for *in vivo* applications. While limitations may constrain further improvements within this narrow SAR, it is noteworthy that our investigation has predominantly focused on just two out of five series. This leaves a significantly untapped reservoir of TRPM4 antagonists, but also potentiators, which could substantially facilitate future advancements in drug development. Recent findings propose compounds that inhibit TRPM4-NMDAR binding as a treatment option for neurodegenerative diseases.^24^ This approach assumes the selective targeting of only the toxic component of NMDAR signaling, preserving synaptic function. However, it remains uncertain whether alterations in NMDAR function resulting from this approach will circumvent the adverse effects observed with previous treatments targeting NMDARs. In comparison, the reported behavioral alterations or other adverse effects in animals treated with meclofenamate,^45^ TRPM4 knockout animals^39^^,^^41^^,^^51^^,^^52^^,^^54^^,^^78^ or animals treated with TRPM4 blocking antibodies^40^ are marginal.

Finally, our *in vitro* pharmacology data and *in vivo* DMPK results suggest low toxicity and minimal off-target effects of our TRPM4 antagonist. This positions them as promising options for treating neurodegenerative diseases and diseases associated with TRPM4 mutations. Our electrophysiological analysis of binding site mutants supports the proposal of a common compound binding site, and the framework presented herein can accelerate the advancement toward clinical applications. The precise activity and on-target structure data generated to date will provide a constructive guidance when optimizing the metabolic stability of these antagonists further.

### Limitations of the study

While our study has identified promising TRPM4 antagonists and provided valuable insights into their binding mechanisms and potential therapeutic applications, some limitations should be noted. Our medicinal chemistry efforts focused primarily on Series 1 and 3 of the five identified lead series. This narrow focus may have resulted in overlooking potential compounds in the other series that may have better pharmacokinetic properties or higher potency. Future studies should investigate these additional series to fully utilize the potential of the entire compound screen. The SAR of Series 1 showed limited tolerance to modifications, particularly within the phenyl moiety, indicating a restricted binding pocket. This limited SAR may complicate further improvements in the pharmacokinetic properties and therapeutic efficacy of the compounds. Our results show that some compounds, such as those of Series 1, exhibit significantly different activities between mouse and human TRPM4. This species-specific activity makes it difficult to transfer preclinical results from mouse models to potential therapies in humans. Further investigations into the causes of these differences are necessary to ensure the efficacy of these compounds in different animal species or warrants humanized TRPM4 animals. *In vivo* pharmacokinetic evaluations revealed low half-lives for the compounds, particularly in the brain. Rapid metabolism may reduce therapeutic efficacy and require frequent dosing, which could limit the applicability of these compounds in the clinical setting. Improving the metabolic stability of these antagonists is crucial before conducting large *in vivo* studies. In summary, while our study provides a solid foundation for the development of TRPM4 antagonists as potential therapeutics for neurodegenerative diseases, addressing these limitations through future research will be critical to realizing their full clinical potential.

## Resource availability

### Lead contact

Further information and request for resources and reagents should be directed to and will be fulfilled by the lead contact, Manuel A. Friese (manuel.friese@zmnh.uni-hamburg.de).

### Materials availability

Plasmids and compounds generated in this study are available upon reasonable request from the corresponding author.

### Data and code availability

• Data: This study did not generate datasets that are publicly available in external repositories. All data supporting the findings of this study are included in the main text and supplemental information.
• Code: This paper does not report original code. The R packages and libraries used for data analysis are listed in the key resources table.
• All other items: Any additional information required to reanalyze the data reported will be shared by the lead contact upon request.

## Acknowledgments

We thank the Friese laboratory for discussions and critical comments. We thank Dr. Sabine Fleischer for her support of this project as scientific coordinator. The work was supported by the 10.13039/501100002347*Bundesministerium für Bildung und Forschung* (NEU^2^ Biopharma 16GW0056 to M.A.F., VIP+ 03VP01752 to M.A.F. and 03VP01751 to P.G. and Target validation 16GW0308K to M.A.F. and 16GW0309 to O.P.) and the 10.13039/501100001659*Deutsche Forschungsgemeinschaft* (FR1720/27-1 to M.A.F.). M.S.W. is funded by the Else Kröner Fresenius Memorial Stipend. R.V. and A.P. were supported by the FWO-Vlaanderen (G051024N) and Bijzonder Onderzoeksfonds – KU Leuven (TRPLe).

## Author contributions

L.B-.L., O.P., P.G., and M.A.F. conceived and supervised the study. L.B-.L. conducted glutamate excitotoxicity experiments, modeling, and data analysis. D.M. performed early assay development. M.S.W. performed TMRE experiments. A.P. performed electrophysiology experiments. R.V. provided expertise and supervision of electrophysiology. C.A., A.E., and S.H. supervised the experiments at Evotec. T.J. supported the study by external mentoring. A.W. supported the project as scientific coordinator. U.H. helped with experiments and contributed to discussions. L.B-.L. and M.A.F. wrote the initial version of the manuscript. O.P., P.G., and M.A.F. funded the study. All co-authors contributed to the editing and discussion of the manuscript and approved the final version.

## Declaration of interests

L.B-.L. and M.A.F. are inventors on a filed patent application covering the therapeutic use of the described compounds to block TRPM4. All other authors declare no conflicts of interest.

## STAR★Methods

### Key resources table

**Table:** 

REAGENT or RESOURCE	SOURCE	IDENTIFIER
**Chemicals, peptides, and recombinant proteins**		

Monosodium Glutamate	Merck	PHR2634
DMEM	Thermo Fisher Scientific	10566016
DMEM/F-12	Thermo Fisher Scientific	10565018
HBSS	Thermo Fisher Scientific	14170112
poly-L-lysine	Sigma-Aldrich	A-003-M
MitoTracker dye	Thermo Fisher Scientific	M7512
FLIPR membrane potential blue dye	Molecular Devices	R8034
TMRE	Abcam	ab113852

**Critical commercial assays**		

RealTime-Glo MT Cell Viability Assay	Promega	G9711
PNGM™ Primary Neuron Growth Medium BulletKit™	Lonza	CC-4461

**Software and algorithms**		

Open Babel library	O’Boyle et al.^81^	v3.1.1
ChemmineR	Cao et al.^82^	v3.17
RCDK	Guha^83^	v3.8.1
PK	Jaki and Wolfsegger^84^	v1.3-6
DoGSiteScorer	Volkamer et al.^85^	
JAMDA	Flachsenberg et al.^86^	
Proteins*Plus*	Schöning-Stierand et al.^87^	
PyMOL	Schrödinger and DeLano^88^	2.5.5

### Experimental model and study participant details

#### Animals

C57BL/6J wild-type mice (The Jackson Laboratory) were kept under specific pathogen-free conditions in the central animal facility of the University Medical Center Hamburg-Eppendorf (UKE) or at Evotec. We used adult mice (6–20 weeks old) from both sexes; mice were sex- and age-matched in all experiments. All animal care and experimental procedures were performed according to institutional guidelines and conformed to the requirements of the German legal authorities.

#### Cell lines

##### HEK293 cells

HEK293 cells parental cells, TRPM4- and TRPM5-expressing HEK293 cells (Anaxon AG, Bern, Switzerland) were cultured in DMEM (Thermo Fisher Scientific, 10566016) supplemented with 10% FCS (PAN, P30-3306) at 37°C and 5% CO_2_. Cells were split every 3 to 4 days at a confluence of approximately 70%.

##### Mouse primary neurons

Primary mouse neuronal cultures were prepared following the methods outlined previously.^26^ Briefly, cortices or hippocampi from C57BL/6J (Jackson Laboratory) E15.5 embryos were isolate and subjected to trypsin digestion for 6 minutes at 37°C. The digestion process was stopped using DMEM/F-12 (Thermo Fisher Scientific, 10565018) supplemented with 10% fetal calf serum (FCS), followed by tissue washing with HBSS (Thermo Fisher Scientific, 14170112). Subsequently, cells were dissociated in PNGM (Lonza, CC-4461). Dissociated cells were seeded at a density of 60,000 cells per cm^2^ onto poly-L-lysine-coated (5 μM; Sigma-Aldrich, A-003-M) well plates or glass cover slips and cultured at 37°C and 5% CO_2_ for a minimum of 14 days before further experimentation without the addition of cytosine arabinoside (AraC) to inhibit glial proliferation. Ethical approvals were obtained from the State Authority of Hamburg, Germany (study approval number: 122/17).

### Method details

#### Small molecule library

We conducted screening for small molecule TRPM4 antagonists using a drug-like and lead-like compound library from Evotec SE, consisting of 256,286 compounds with high chemical diversity of compounds. All compounds were dissolved in DMSO at a stock concentration of 20 mM and were applied to reach a final concentration of 10 μM.

#### FLIPR assay

HEK293 parental cells and HEK293 cells stably expressing human TRPM4 or TRPM5 (Anaxon AG, Bern, Switzerland) were seeded at 10,000 cells per well in 50 μl growth medium supplemented with 9% FCS in 384-well plates. Next day, cells were washed once in FLIPR Membrane Potential Assay Kit (Molecular Devices). Subsequently, the buffer was aspirated to a residual volume of 25 μl before addition of 25 μl FLIPR membrane potential (FMP) blue dye (Molecular Devices, R8034). Cells were incubated for 60 min at 37°C before placement into the FLIPR Tetra High-Throughput Cellular Screening System (Molecular Devices). For the first FLIPR-read, 5 μl of the test compounds, the positive control TRPM4 antagonist 9-phenanthrol or FAA, vehicle control DMSO 0.25% or activation control ionomycin were added and fluorescence was measured for 140 seconds. Finally, 5 μl ionomycin in a final concentration of 1 μM was added to induce intracellular Ca^2+^ rise that activates TRPM4 and the second FLIPR-read was obtained for an additional 140 seconds.

#### QPatch assay

QPatchHTX, an automated electrophysiology platform, was used in single hole mode. One day before use human TRPM4- or TRPM5-expressing HEK293 cells were seeded in a T75 flask at a density of 53k cells per cm^2^ and at passages < 23. For QPatch assays, cells were harvested and re-suspended in SFM (Gibco, 11686029). The extracellular solution was (in mM): CaCl_2_ (7), MgCl_2_ (2), KCl (5), HEPES (10), NaCl (70), NMDG (70); pH 7.4 with NaOH. The intracellular solution was (in mM): HEPES (10), NMDG (100), NaCl (50), EGTA (20), MgCl_2_ (1), CaCl_2_ (21); pH 7.2 with HCl. After break-in, channels were consistently open and current through the channels could be observed at a voltage ramp from –100 mV to +100 mV over 500 ms. This protocol was repeated every 7 seconds. After saline and vehicle application, the channel was desensitized and residual current was used as baseline. FFA was used as reference compound and applied cumulatively with each concentration incubating at a minimum of 20 voltage protocol runs (140 seconds) with the current after addition of FFA being calculated as full block. Compounds were prepared from 20 mM stocks in DMSO and first measured at 0.3, 3 and 30 μM concentrations to establish 3-point IC50 curves. If compounds were potent, concentrations were adjusted to 0.003, 0.03, 0.3, 3 and 30 μM to obtain precise IC50 values. Each compound was measured at least 3 time independently.

#### Excitotoxicity assays

Excitotoxicity assays were performed using primary mouse neuronal cultures between 14 and 17 days *in vitro* (DIV). Cell viability was measured using RealTime-Glo MT Cell Viability Assay (Promega, catalog no. G9711) according to the manufacture protocol. Compounds were co-administered with RealTime-Glo reagents, and luciferase measurements were conducted using the Tecan-Spark M10 microplate reader with environmental control set to 37°C and 5% CO_2_. Neuronal cultures were stimulated with either glutamate or vehicle five hours after compound application and monitored for an additional 20 hours. To adjust for inter-well variability, arbitrary light units were normalized to a steady signal prior to glutamate addition for each well. Toxicity was calculated relative to the minimal cell viability at 15 hours post glutamate stimulation and cell viability of the PBS vehicle control.

#### TMRE assay

Primary mouse neuronal cultures were grown on μ-Dish 35 mm Quad dishes (ibidi, 80416) and were treated after 15–17 d.i.v. with either vehicle or CMP312 for 30 minutes. Subsequently 50 μM glutamate was applied for in total 2 hours. Then, 20 nM TMRE dye (Abcam, ab113852), 500 nM MitoTracker dye (ThermoFisher, M7512), and 1 μM Hoechst 33342 were added after 1.5 hours glutamate stimulation for 30 minutes. Afterwards cells were washed two times in pre-warmed medium and were imaged immediately in preconditioned medium using a confocal microscope (Zeiss, LSM700) with 20-fold magnification and controlled atmosphere conditions (37°C, 5% CO_2_). Imaging time was less than 10 minutes per condition. We quantified the total MFI of the MitoTracker and TMRE dyes per cell. At least 50 cells per condition were quantified and the mean ratios of TMRE to Mitotracker per independent experiments were reported.

#### Hepatocyte clearance

Cryopreserved hepatocytes were exposed to test Compounds at a concentration of 1 μM for 120 minutes. Samples were collected at various time points, and percent remaining of test compound was determined using mass spectrometry (LC-MS/MS) to calculate hepatic clearance rate (L/h/kg).

#### Microsome clearance

Human or mouse liver microsomes were incubated with the test compound at a concentration of 1 μM for 45 min in the presence of cofactor (NADPH in excess). Samples were collected at various time points, and % remaining of test compound was determined using mass spectrometry (LC-MS/MS) to calculate hepatic clearance rate (L/h/kg).

#### CYP inhibition assay

The half-maximal inhibitory concentration (IC50) for cytochrome P450 (CYP) enzymes was determined using an *in vitro* enzyme inhibition assay. Recombinant CYP enzymes or human liver microsomes from BD Gentest, in 0.1 M phosphate buffer pH 7.4, containing native CYP enzymes were incubated with the test compound at various concentrations (1, 3, 10 and 30 μM) and containing 0.5% DMSO and 0.1% BSA final concentrations. 1 mM of NADPH cofactor was added and the reaction was started by a rapid increase of temperature until 37°C. The IC50 value was determined as the concentration of the test compound required to inhibit 50% of the enzyme activity by measuring metabolite formation and was calculated using appropriate dose-response curve fitting methods.

#### Caco-2 permeability assay

Caco-2 permeability was evaluated using a cell-based assay with Caco-2 cells cultured on permeable membrane inserts. The test compound was added at 10 μM to the apical (donor) compartment, and samples were collected from the basolateral (receiver) compartment at 120 min. The test compound was also added at 10 μM to the basolateral (donor) compartment, and samples were collected from the apical (receiver) compartment at 120 min. The amount of the compound in both compartments was measured using mass spectrometry (LC-MS/MS). The permeability coefficient (Papp, nm/s) apical to basolateral and basolateral to apical, and an efflux ratio were calculated.

#### PAMPA

Parallel Artificial Membrane Permeability Assay (PAMPA) was performed using artificial lipid membrane layers to assess passive permeability of the test compound. Test compounds were diluted to 200 μM in system buffer at pH 5.0, 6.2 and 7.4. The solutions were filtered and 150 μL transferred to a High Sensitivity 96 well UV plate. This was analysed by UV as the reference plate. A 200 μL sample of the 200 μM solution was transferred to the ‘donor’ plate of the PAMPA pIon sandwich plate system. 200 μL of acceptor sink buffer was transferred to the ‘acceptor’ plate which had been previously treated with GIT-O lipid solution across the well filter. Donor and acceptor plates were sandwiched and kept in a humid environment at room temperature for 16 hours. On completion of the incubation the donor and acceptor plates were separated. 150 μL of solution was transferred from each of the donor and acceptor PAMPA sandwich plates into High Sensitivity 96 well UV plates for analysis by UV. at different time points. The concentration of the compound in the acceptor compartment was determined using mass spectrometry. The permeability coefficient (Papp) was calculated based on the rate of appearance of the compound in the acceptor compartment (nm/s).

#### *In vitro* pharmacology

For *in vitro* pharmacological profiling and determination of off target effects compound were screened at a 5 μM in duplicate for their potential to bind to a broad panel of receptors, enzymes, and ion channels on a commercially available platform (Safetyscreen 44 Panel; Eurofins Cerep, Le Bois l'Evêque, B.P. 30001, 86 600 Celle l'Evescault, France), Study ID FR095-0009609).

#### *In vivo* pharmacokinetics

Three female C57BL/6J (Jackson Laboratory) mice per group and time point were injected intravenously or intraperitoneally using the following concentrations per bodyweight: CMP354, CMP343 and CMP233 i.v. 3 mg kg^–1^, i.p. 10 mg kg^–1^; CMP157 i.v. 2.5 mg kg^–1^, i.p. 5 mg kg^–1^. Animals were sacrificed after 5, 15, 60, 120, 240, 420 or 1440 minutes post injection and compound concentrations were determined by mass spectrometry in plasma, heart and brain. Half-life and bioavailability were calculated by noncompartmental analysis using the R package PK^84^ (v1.3-6).

#### Molecular biology

Human *TRPM4* (UniProtKB: Q8TD43-1) and mouse *Trpm4* (UniProtKB: Q7TN37-1) were amplified via PCR and inserted into the lentiviral vector pFUGW (Addgene: 14883), which harbors a custom multiple cloning site and a C-terminal eGFP tag, using the restriction enzymes NheI and Pfl32II. Additionally, human TRPM4 was inserted into the pcDNA3.1 plasmid using the same restriction enzymes. TRPM4 mutants were generated by site-directed mutagenesis using the Q5 Site-Directed Mutagenesis Kit (Promega, E0554S).

#### Electrophysiology

Patch clamp recordings were conducted using an EPC10 patch clamp amplifier and Patchmaster software (HEKA, Germany). An Ag/AgCl wire served as the reference electrode in all experiments. Patch pipettes, pulled from borosilicate capillary tubes using a DMZ universal puller, exhibited final resistances ranging from 2 to 4 MΩ. Liquid junction potential was compensated before achieving gigaseal formation. All experiments were conducted at room temperature. Whole-cell currents were recorded in transiently transfected HEK293 cells expressing either wildtype human TRPM4, TRPM4-L907A, or TRPM4-S924A. Recordings involved a 400 ms increasing voltage ramp from –100 mV to +100 mV, initiated from a holding potential of 0 mV. The interval between each sweep was 2 seconds, with data sampled at 5 kHz. The extracellular solution composition was as follows (in mM): NaCl (156), HEPES (10), glucose (10), CaCl_2_ (1.5), and MgCl_2_ (1), pH 7.4. To identify TRPM4 currents, extracellular Na^+^ was replaced with equimolar NMDG^+^. Inside-out currents were recorded from membrane patches excised from transiently transfected HEK-293T cells expressing either wildtype human TRPM4, TRPM4-L907A, TRPM4-S924A, or wildtype mouse TRPM4. Similar to whole-cell recordings, a 400 ms increasing voltage ramp from –100 mV to +100 mV was applied from a holding potential of 0 mV. The interval between sweeps was 2 seconds, and data were sampled at 5 kHz. The pipette solution comprised (in mM): NaCl (156), HEPES (10), glucose (10), CaCl_2_ (1.5), and MgCl_2_ (1), pH 7.4, while the intracellular solution contained (in mM): NaCl (20), NaAsp (120), HEPES (10), and MgCl_2_ (1), pH 7.2. Transiently transfected HEK-293T cells were sealed, and membrane patches were excised using the intracellular solution, supplemented with 10 mM EGTA. TRPM4 currents were activated by 500 μM CaCl_2_ and deactivated by 10 mM EGTA, both added to the intracellular solution. Ca^2+^ activation curves were fitted using a likelihood maximization algorithm to estimate the parameters for the sigmoidal fitting curves y-maximum, slope and midpoint as part of the R package sicegar (V0.2.4).

#### Multidimensional scaling

Compounds were canonicalized using the Open Babel library^81^ and R package ChemmineR (v3.17). Extended molecular fingerprints of the compounds and Tanimoto similarity matrices were calculated using R package RCDK (v3.8.1).

#### Modeling and compound docking

The human TRPM4 structure in a calcium-bound state (PDB:6bqv) and the zebrafish TRPM5 in a calcium and NDNA-bound state (PDB:7mbv) were aligned using helices S3, S4 and S5’ as reference. Alignments and visualization were performed using the PyMol software. Identification of druggable cavities and docking of compounds were carried out using DoGSiteScorer^85^ and JAMDA,^86^ respectively, as provided by the Proteins*Plus* modeling tools.^87^

### Quantification and statistical analysis

The statistical analyses applied are detailed in the respective legends to the figures and include the number of biological replicates (N) and the statistical test used. Significant results are indicated by ∗*P* < 0.05, ∗∗*P* < 0.01, ∗∗∗*P* < 0.001.
