# Supplementary material for: Identification and development of TRPM4 antagonists to counteract neuronal excitotoxicity
Source: iScience. 2024 Nov 19;27(12):111425. doi: 10.1016/j.isci.2024.111425 (PMC11648915; doi:10.1016/j.isci.2024.111425)
Supplement: Document S1. Figures S1–S10 [file mmc1.pdf]

iScience, Volume *27*

Supplemental information

Identification and development of TRPM4 antagonists to counteract neuronal excitotoxicity

Lars Binkle-Ladisch, Andy Pironet, Andrea Zaliani, Chantal Alcouffe, Daniel Mensching, Undine Haferkamp, Anne Willing, Marcel S. Woo, Alexandre Erdmann, Timm Jessen, Stephen D. Hess, Philip Gribbon, Ole Pless, Rudi Vennekens, and Manuel A. Friese

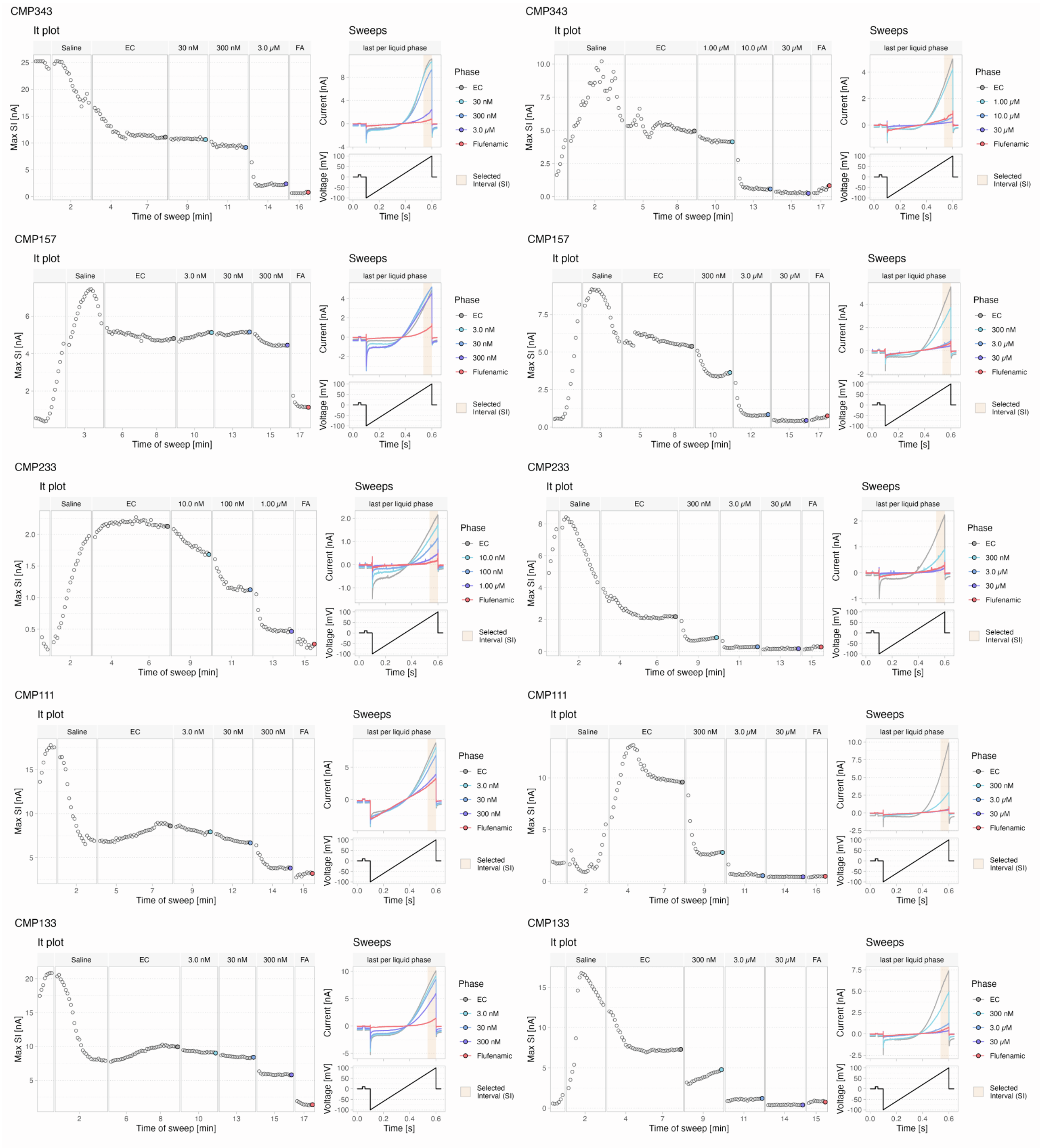

Figure S1. Exemplary traces of QPatch measurements, related to Figure 1.

I-t-plots and sweep traces from representative cells treated with all five lead compounds. After an initial break-in and equilibration phase with saline and extracellular solution (EC), the compounds were applied additively at the indicated concentrations. The example traces shown are from the final sweep of each treatment phase.

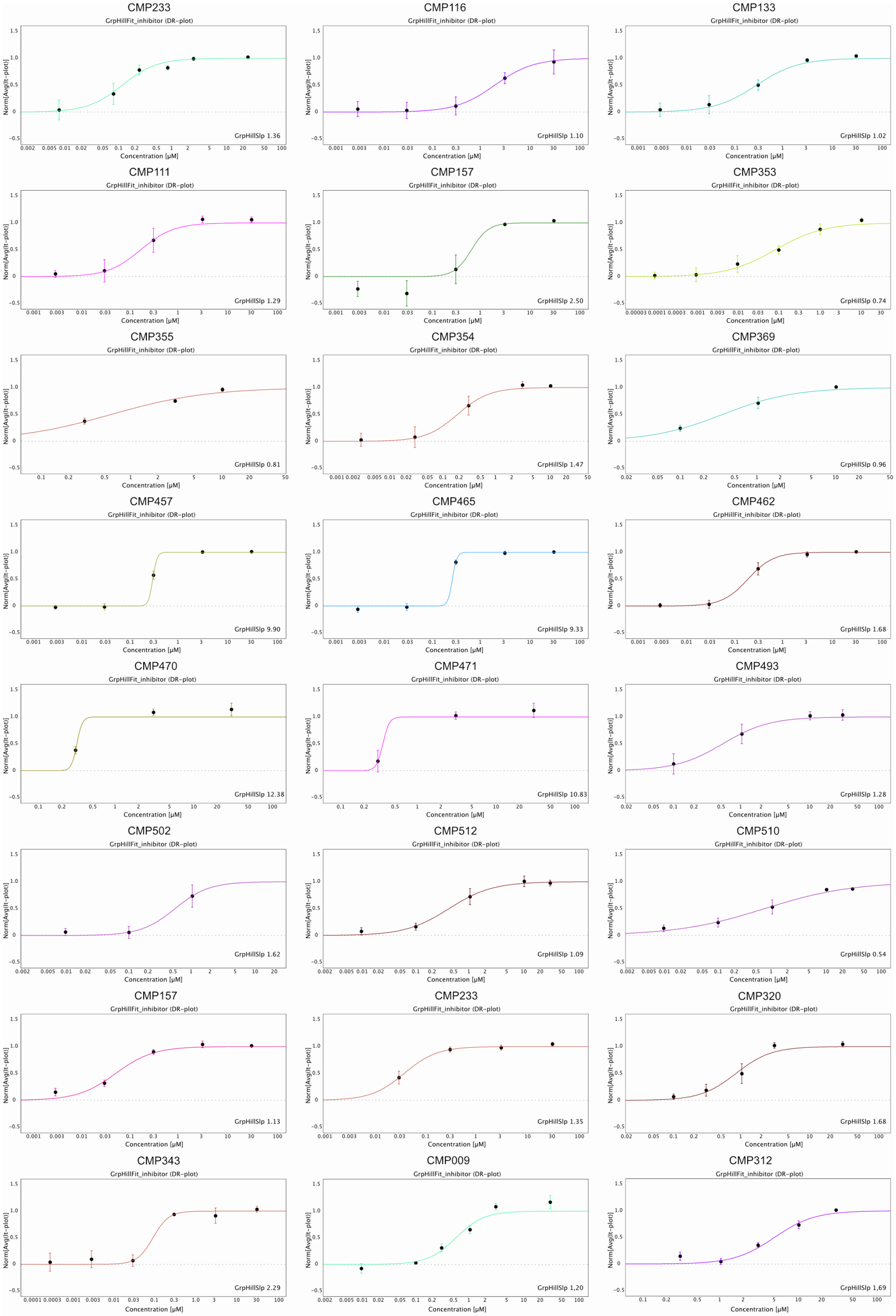

Figure S2. Dose-response curves and Hill fittings of TRPM4 antagonists, related to Figures 1 and 2.
Dose-response curves and Hill fittings are shown for all TRPM4 antagonists, with the indicated slope (Hill slope, HillSlp).

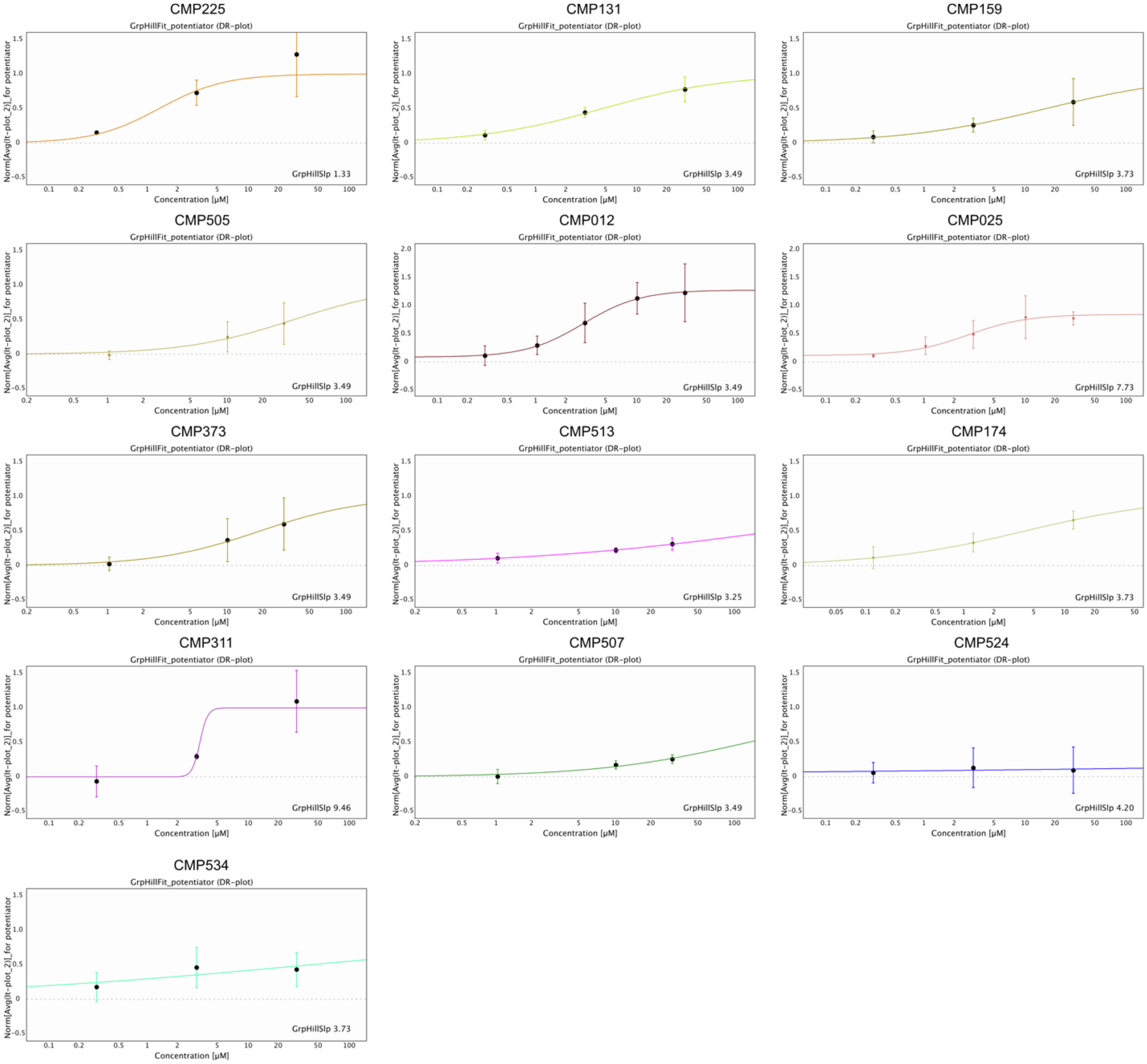

Figure S3. Dose response curves and Hill fittings of TRPM4 potentiators, related to Figures 1 and 2.
Dose-response curves and Hill fittings are shown for all TRPM4 antagonists, with the indicated slope (Hill slope, HillSlp).

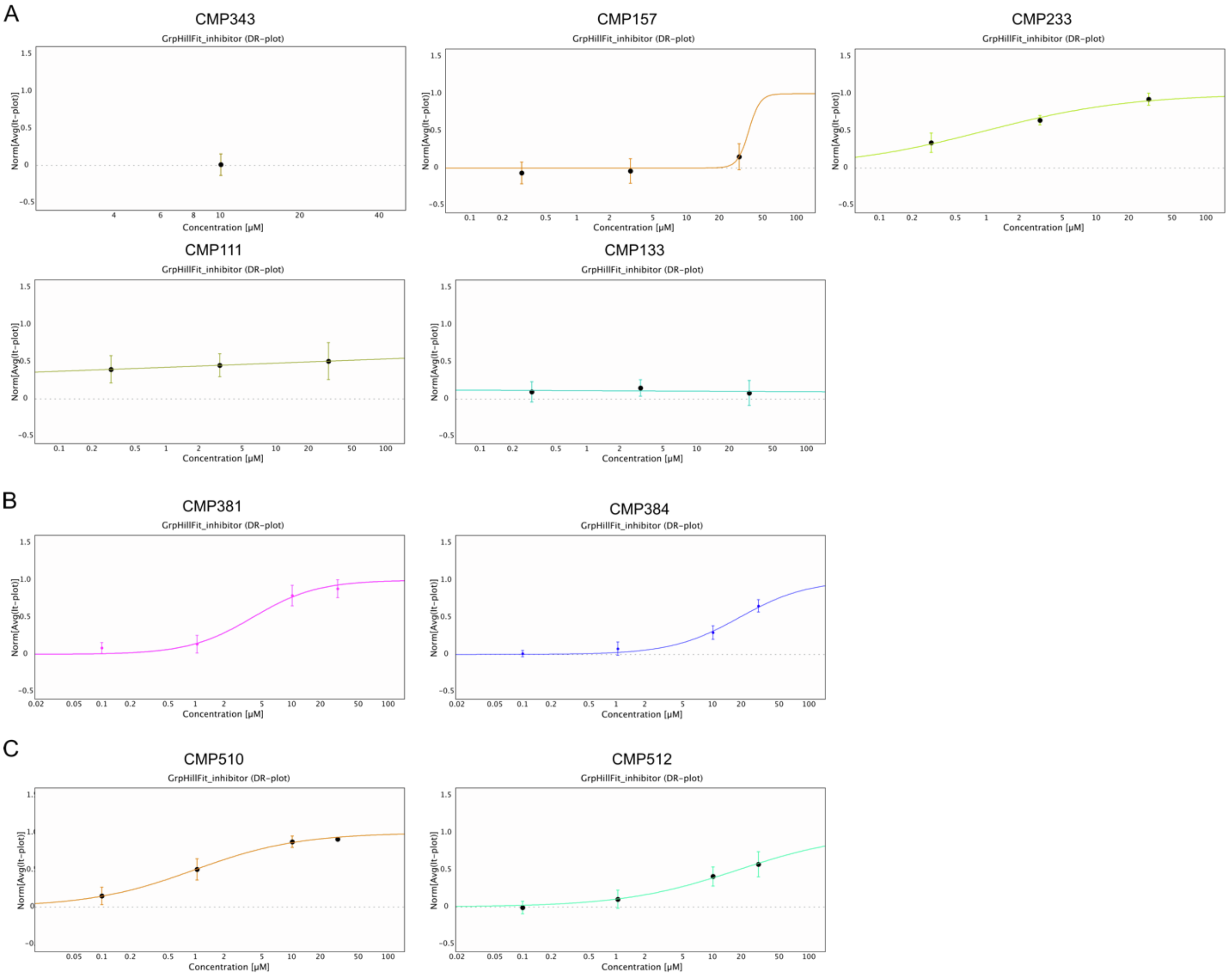

Figure S4. Dose-response curves and Hill fittings of TRPM4 antagonists on TRPM5, related to Figure 2.
(A) Dose-response curves and Hill fittings of TRPM4 lead compounds from all five series on TRPM5.
(B) Dose-response curves and Hill fittings of TRPM4 Series 1 antagonists, showing TRPM5 activity identified during medicinal chemistry optimization.
(C) Dose-response curves and Hill fittings of TRPM4 Series 3 antagonists, showing TRPM5 activity identified during medicinal chemistry optimization.

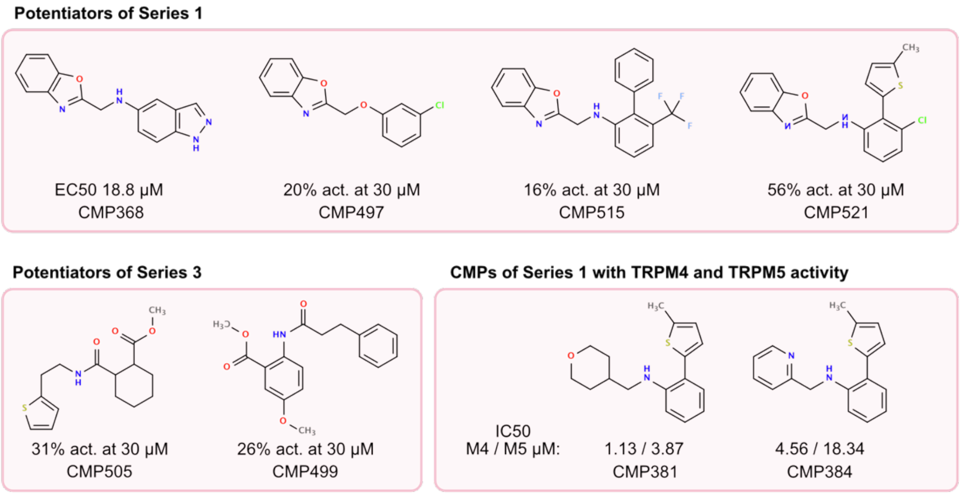

Figure S5. Potentiators of Series 1 and 3 and antagonists of Series 1 with TRPM5 activity, related to Figure 2. Potentiators of Series 1 and 3 that have been discovered during the medical chemistry optimization of the series. Except for CMP368, activity is indicated as percent activation of TRPM4 at the highest measured compound concentration. TRPM4 antagonists discovered during the medical chemistry optimization of the Series 1 with TRPM5 activity.

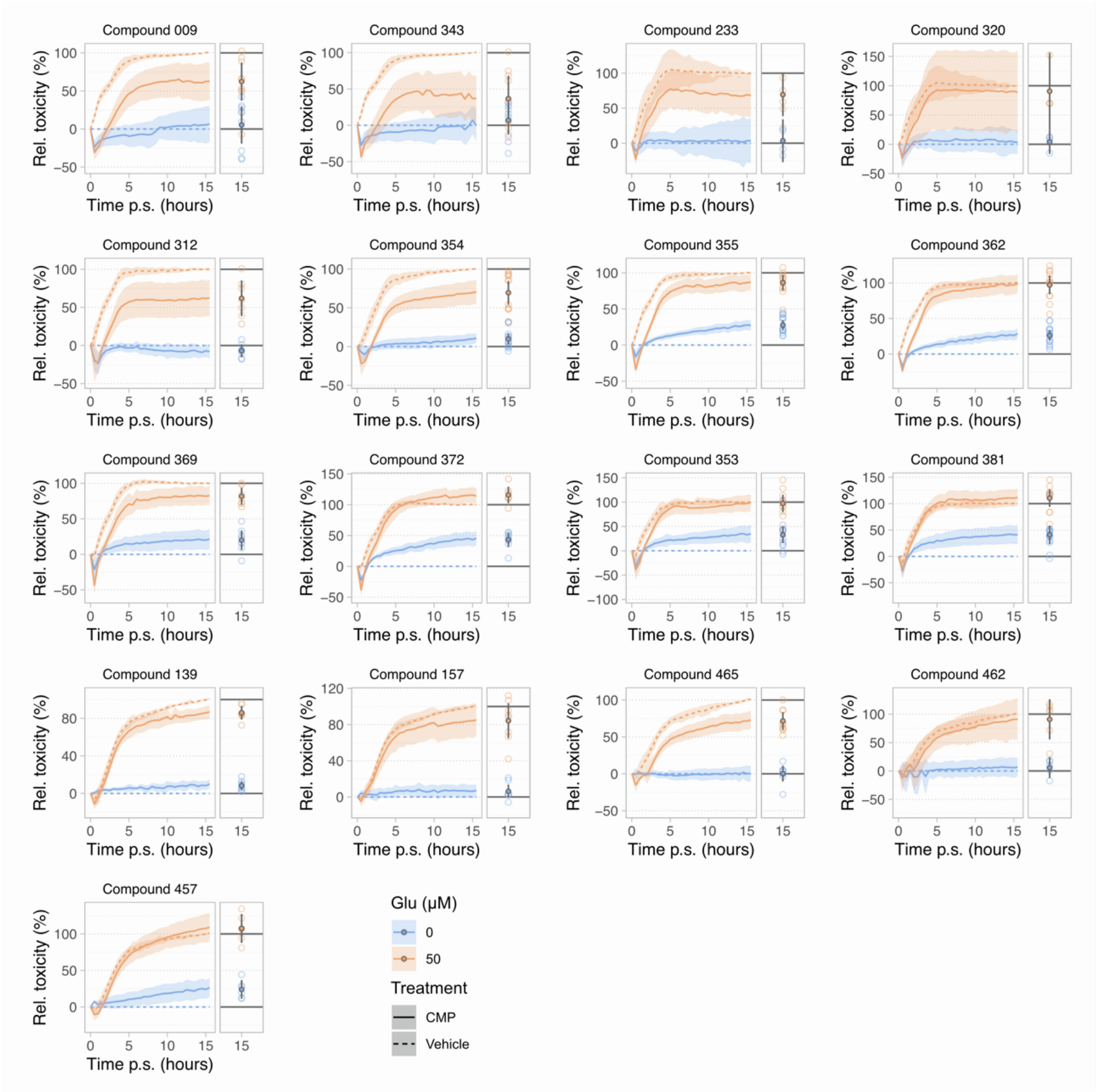

Figure S6. Neuroprotective *in vitro* evaluation, related to Figure 3.
In vitro evaluation of compound activity on glutamate-induced excitotoxicity in mature primary neuronal cultures. Time course (left panel) and 15 hours virtual endpoint (right panel). Data are represented as mean with 95% confidence interval.

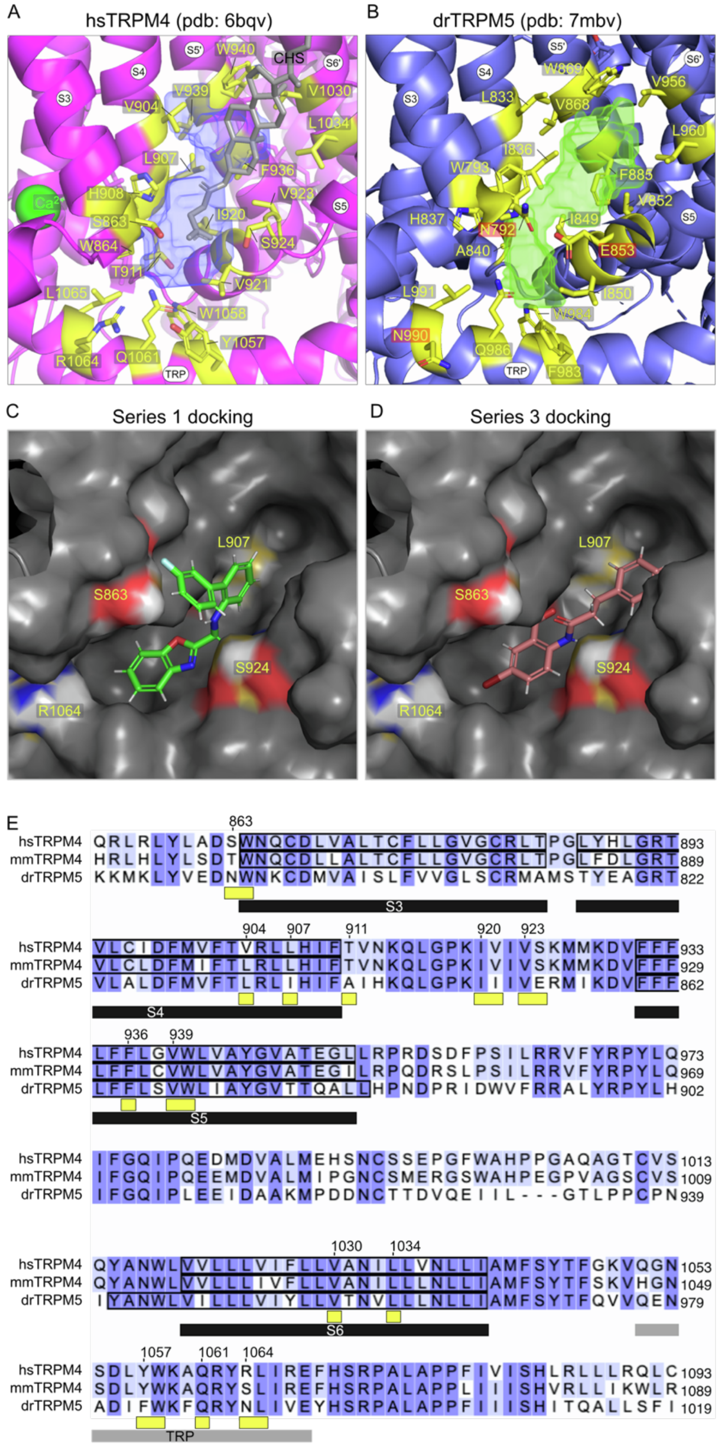

Figure S7. Proposed compound binding site, related to Figure 4.

(A, B) The proposed compound binding site in hsTRPM4 (A) and the homologous site in drTRPM5 (B) are depicted. Transmembrane helices S3-S5, the S4-S5 linker, TRP domain, and helices S5' and S6' from the neighboring TRPM4 subunit, along with the amino acids lining the binding pocket, are labeled. Predicted pocket volumes are represented as colored clouds, with major differentiating amino acids between hsTRPM4 and drTRPM5 highlighted in red.

(C, D) Docking of Series 1 compound CMP343 (C) and Series 3 compound CMP233 (D) to the proposed binding site.

(E) Sequence alignment of hsTRPM4, mmTRPM4 and drTRPM5 in the region of the proposed binding site. Relevant amino acids from A are labeled in yellow, transmembrane helices are labeled in black and the TRP domain in gray.

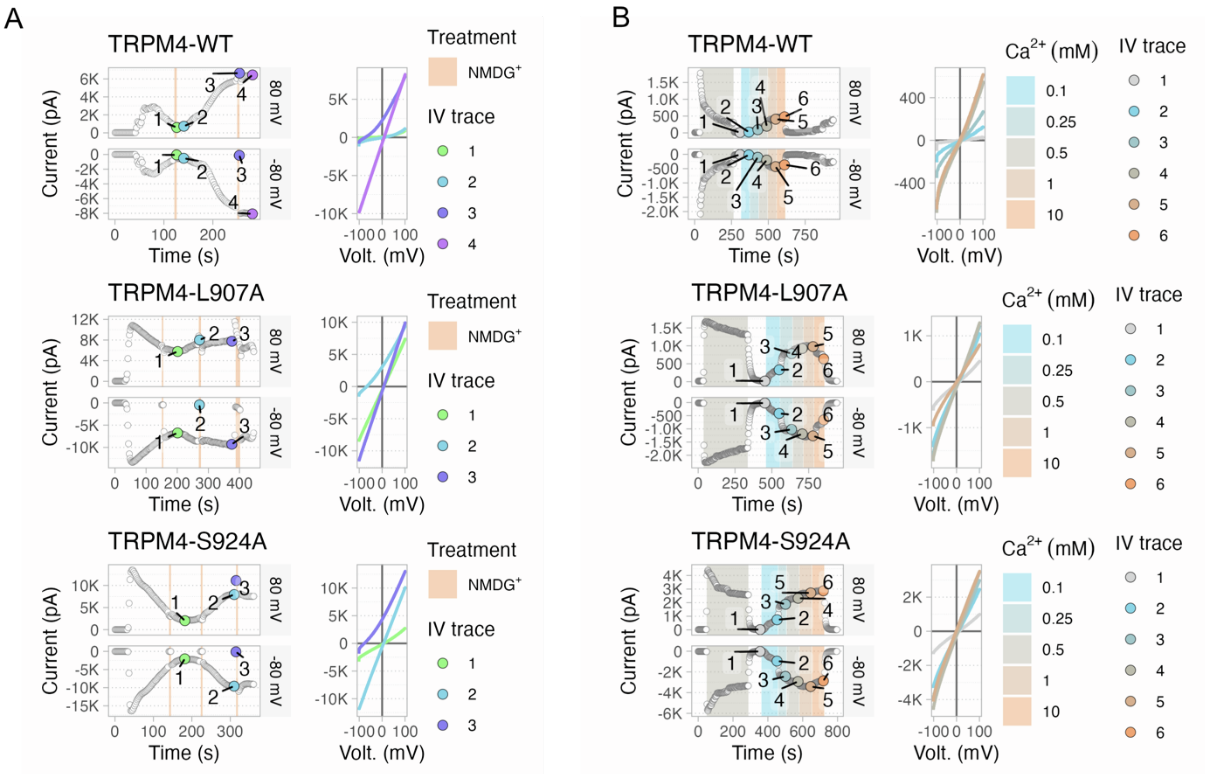

Figure S8. Electrophysiological characterization of TRPM4 mutants, related to Figure 4.

(A) Whole cell voltage-clamp analysis of basic electrophysiological properties of HEK293T cells expressing wild-type or mutant TRPM4. Representative time course of current level at –80 and +80 mV measured in either hsTRPM4-WT, hsTRPM4-L907A or hsTRPM4-S924A expressing HEK293T cell using the whole cell patch clamp technique. TRPM4 currents were activated upon patch rupture by 100 µM $CaCl_2$, loaded in the pipette solution. Extracellular Na^+ was replaced by equimolar $NMDG^+$ to identify TRPM4 currents. Individual current-to-voltage relationship measured in either the same hsTRPM4-WT, hsTRPM4-L907A or hsTRPM4-S924A expressing HEK293T cell are shown next to the representative time course.

(B) Assessment of Ca^{2+} sensitivity of wild type and mutant TRPM4. Representative time course of current level at –80 and +80 mV measured in either a hsTRPM4-WT, hsTRPM4-L907A or hsTRPM4-S924A containing membrane patch using the inside-out patch clamp technique. TRPM4 currents were activated by 500 µM Ca^{2+} and deactivated by 10 mM EGTA. Individual current-to-voltage relationship measured in either the same hsTRPM4-WT, hsTRPM4-L907A or hsTRPM4-S924A expressing HEK-293T cell are shown next to the representative time course.

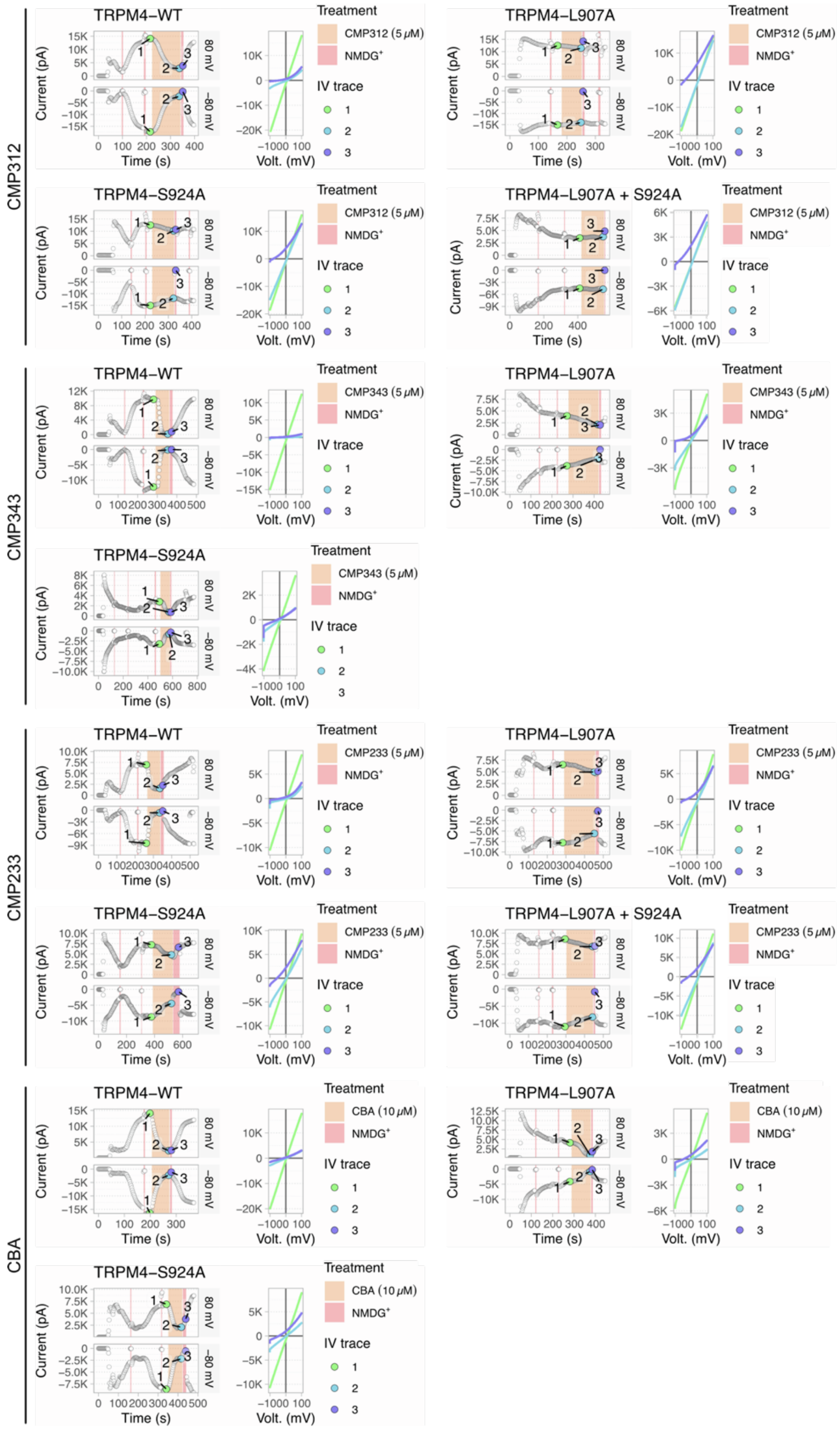

Figure S9. Electrophysiological analysis of proposed compound binding site, related to Figure 4.
Whole cell voltage-clamp measurements of relative inhibition of wild-type and mutant TRPM4 by Series 1 compounds (CMP312, CMP343), Series 3 compound (CMP233) and CBA. Representative time course of current levels at –80 and +80 mV measured in either hsTRPM4-WT, hsTRPM4-L907A or hsTRPM4-S924A expressing HEK293T cell using the whole cell patch clamp technique. TRPM4 currents were activated upon patch rupture by 100 μM $CaCl_2$, loaded in the pipette solution. Extracellular Na^+ was replaced by equimolar $NMDG^+$ to identify TRPM4 currents. Individual current-to-voltage relationship measured in either the same hsTRPM4-WT, hsTRPM4-L907A or hsTRPM4-S924A expressing HEK293T cell are shown next to the representative time course.

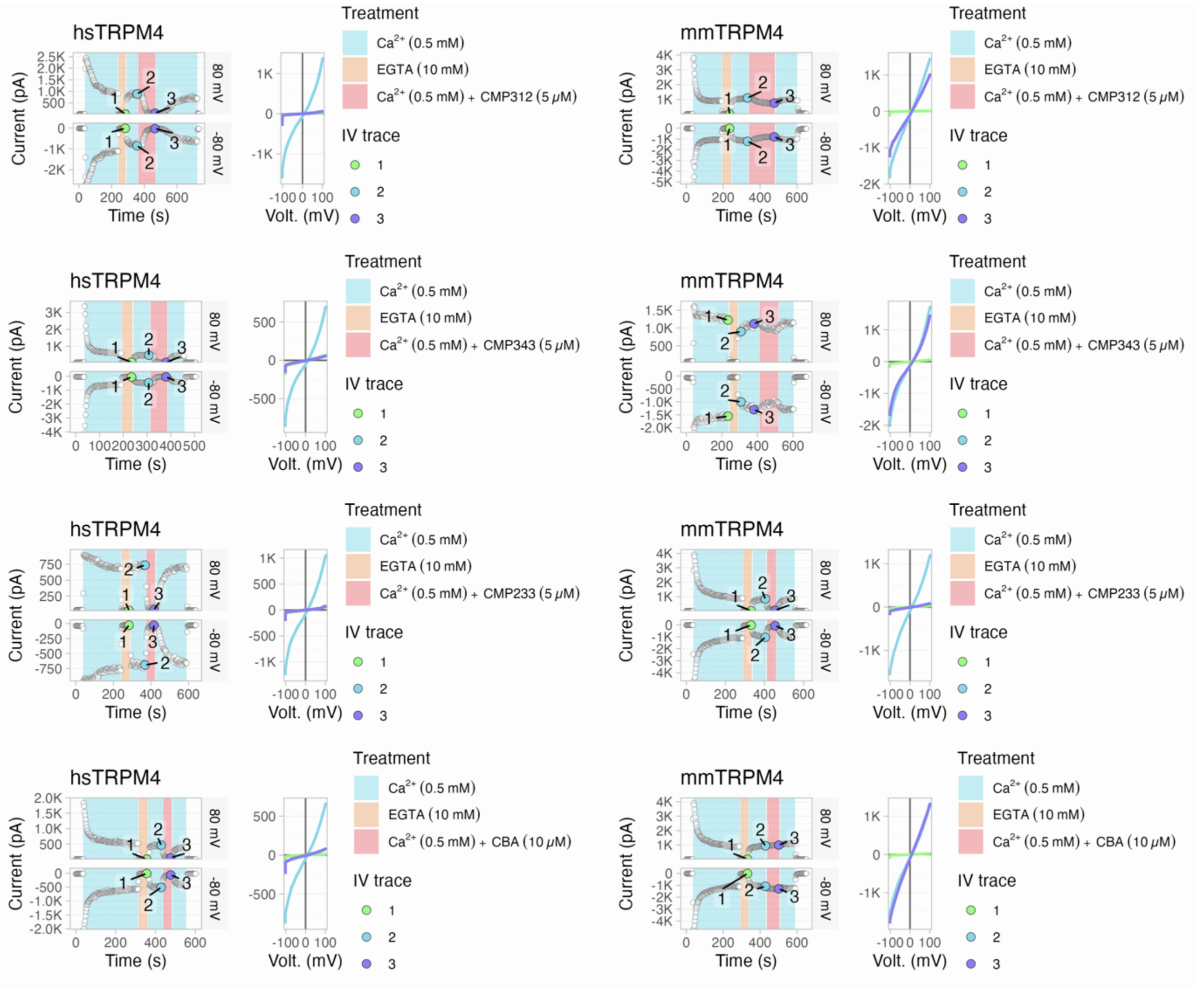

Figure S10. Electrophysiological comparison of human and mouse TRPM4, related to Figure 4.

Whole cell voltage-clamp measurements of relative inhibition by CMP343, CMP233 and CBA comparing mouse (mm) and human TRPM4. Representative time course of current levels at –80 and +80mV measured in either a hsTRPM4 or mmTRPM4 containing membrane patch using the inside-out patch clamp technique. TRPM4 currents were elicited by 500 μM Ca^{2+} and deactivated by 10 mM EGTA. Individual current-to-voltage relationship measured in either the same hsTRPM4 or mmTRPM4 expressing HEK293T cell are shown next to the representative time course.
